# Horizontal transmission of gut microbiota attenuates mortality in lung fibrosis

**DOI:** 10.1172/jci.insight.164572

**Published:** 2023-11-28

**Authors:** Stephen J. Gurczynski, Jay H. Lipinski, Joshua Strauss, Shafiul Alam, Gary B. Huffnagle, Piyush Ranjan, Lucy H. Kennedy, Bethany B. Moore, David N. O’Dwyer

**Affiliations:** 1 Department of Microbiology and Immunology and; 2 Division of Pulmonary and Critical Care Medicine, Department of Internal Medicine, University of Michigan Medical School, Ann Arbor, Michigan, USA.; 3 Department of Molecular, Cellular and Developmental Biology, University of Michigan, Ann Arbor, Michigan, USA.; 4 Unit for Laboratory and Animal Medicine, Office of Research, University of Michigan Medical School, Ann Arbor, Michigan, USA.

**Keywords:** Microbiology, Pulmonology, Adaptive immunity, Fibrosis

## Abstract

Pulmonary fibrosis is a chronic and often fatal disease. The pathogenesis is characterized by aberrant repair of lung parenchyma, resulting in loss of physiological homeostasis, respiratory failure, and death. The immune response in pulmonary fibrosis is dysregulated. The gut microbiome is a key regulator of immunity. The role of the gut microbiome in regulating the pulmonary immunity in lung fibrosis is poorly understood. Here, we determine the impact of gut microbiota on pulmonary fibrosis in substrains of C57BL/6 mice derived from different vendors (C57BL/6J and C57BL/6NCrl). We used germ-free models, fecal microbiota transplantation, and cohousing to transmit gut microbiota. Metagenomic studies of feces established keystone species between substrains. Pulmonary fibrosis was microbiota dependent in C57BL/6 mice. Gut microbiota were distinct by β diversity and α diversity. Mortality and lung fibrosis were attenuated in C57BL/6NCrl mice. Elevated CD4^+^IL-10^+^ T cells and lower IL-6 occurred in C57BL/6NCrl mice. Horizontal transmission of microbiota by cohousing attenuated mortality in C57BL/6J mice and promoted a transcriptionally altered pulmonary immunity. Temporal changes in lung and gut microbiota demonstrated that gut microbiota contributed largely to immunological phenotype. Key regulatory gut microbiota contributed to lung fibrosis, generating rationale for human studies.

## Abstract 2

Gut microbiota-regulated immune responses modify pulmonary fibrosis.

## Graphical abstract

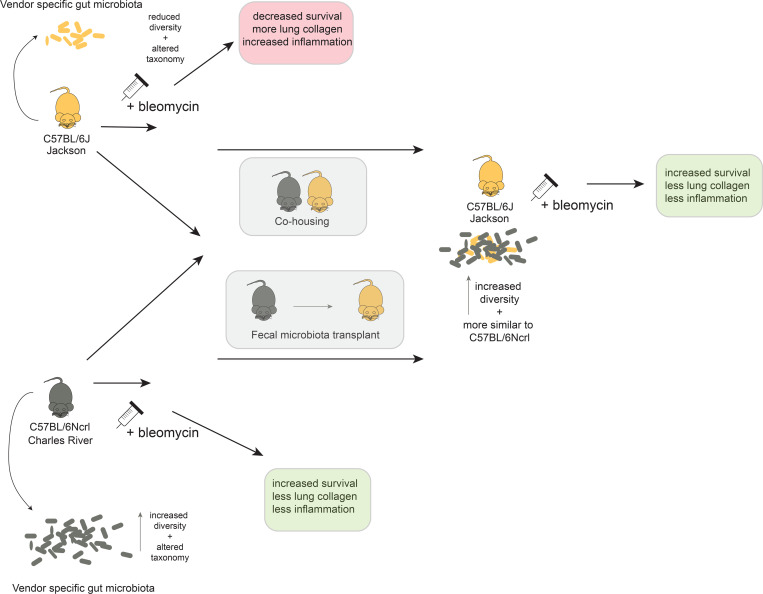

## Introduction

Gut microbiota regulate systemic immunity and influence many aspects of human health (1). Recent work has established a causal role for regulatory gut microbiota in multiple human diseases and demonstrated that gut microbiota are heritable, are shaped by environmental exposures, and are key modifiers of immune cell dynamics (2, 3). These effects are thought to be largely achieved through known mechanisms such as altered innate immune cells, altered inflammatory cytokine responses, and reprogramming of adaptive immunity, in addition to unknown factors (4–6). Key studies have identified a role for gut microbiota in regulating the pulmonary response to infection, and gut microbiota are key variables in determining the myeloid cell pool and accumulation of pulmonary leucocytes in lung injury (7, 8). Recent work in acute lung injury models is controversial, with studies both in support of and against a causal role for gut microbiota or lung microbiota in modifying experimental outcomes (6, 9–11).

Pulmonary fibrosis is a chronic progressive lung disease that results from sequelae of acute lung injury and commonly results in death (12). The pathogenesis of pulmonary fibrosis is poorly understood, no cure exists, and current therapies do not reverse or arrest lung fibrosis (12). A new paradigm is required to understand this heterogenous disease. Previous work has established a role for lung microbiota in the pathogenesis of pulmonary fibrosis, with reported correlations between lung microbiota, alveolar immunity, inflammation, and clinical outcomes in patients with pulmonary fibrosis (9, 13, 14). This supports key interactions between host immunity and mucosal microbiota in the fibrotic lung. We and others have identified dysregulated pulmonary immunity as a key contributor to progressive pulmonary fibrosis (15–18). Gut microbiota are well-established regulators of pulmonary immunity. However, the role of the gut microbiome in lung fibrosis is poorly understood.

Establishing causal associations between gut microbiota and disease is challenging. Previous work has demonstrated a role for vendor-specific commensal microbiota in generating distinct immune profiles associated with outcomes in gastrointestinal disease supporting causal immune cell associations (19). Fecal microbiota transplantation is a method for transferring microbiota from one host to another to study causality but is not standardized in mice, has limitations, and risks poor reproducibility (20). The predominant mechanism of gut microbiota transmission in mammals is vertical through ancestry (21). Experimental mice bred and housed in different barrier facilities will display distinct mucosal microbiota through divergent ancestry (22). However, the horizontal transmission of gut microbiota in mice through bedding and coprophagia may be a more reproducible method for testing causal associations between microbiota and disease (22, 23). In humans gut microbiota are modified by cohabitation and environment (3). The impact of cohousing and horizontal transmission on experimental outcomes in lung disease is not known and represents a major gap in the field.

Here, we hypothesized that distinct gut microbial communities would causally associate with altered pulmonary immune profiles and lung fibrosis. We show that horizontal transmission results in transfer of candidate regulatory taxa from one host to a susceptible host, resulting in protection from lung injury and fibrosis. Thus, we establish a role for regulatory gut microbiota in lung injury and fibrosis, creating a new paradigm for this devastating disease.

## Results

### Lung fibrosis after lung injury is microbiota dependent.

**Figure 1:**
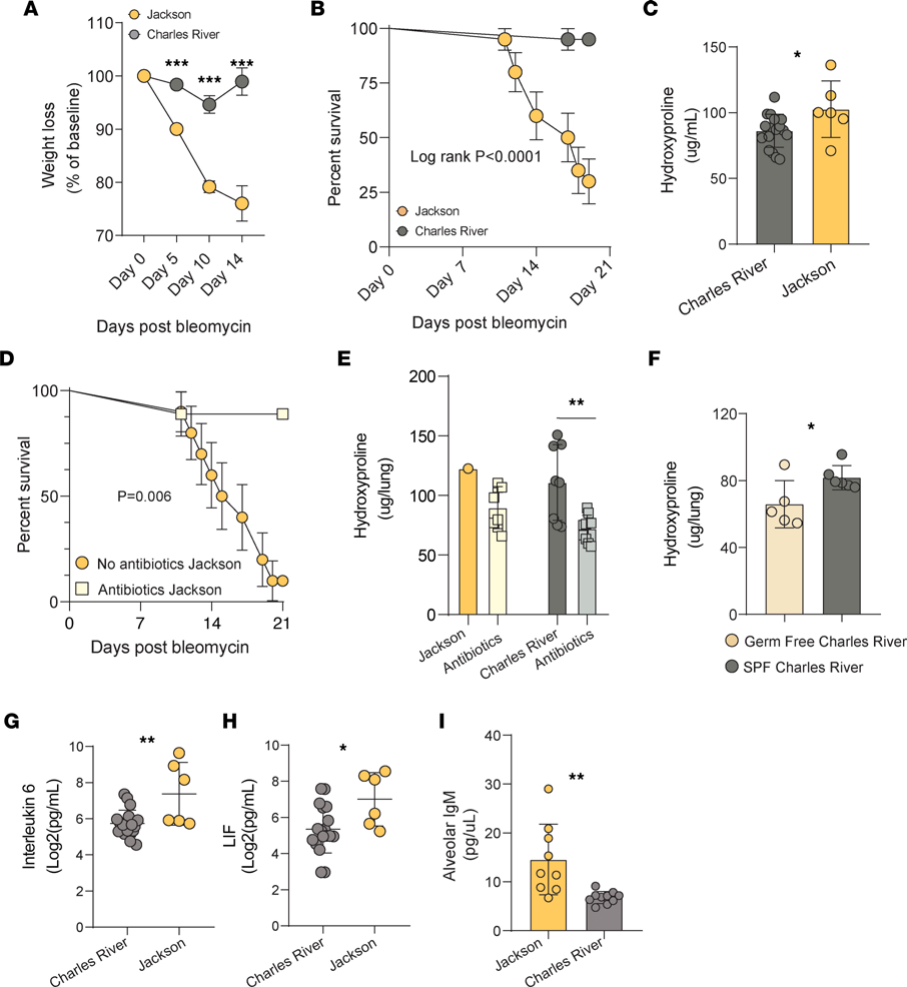
Lung fibrosis is heterogenous and microbiota dependent in substrain C57BL/6 mice. Here, we examined lung fibrosis in C57BL/6 substrain experimental mice (C57BL/6J, JAX, and C57BL/6NCrl, CR). (**A**) JAX mice are more susceptible to weight loss after acute lung injury from bleomycin compared with CR mice. (**B**) JAX mice exhibit increased mortality after bleomycin compared with CR mice (log rank *P* < 0.0001) (JAX mortality 70%, CR mortality 5%). (**C**) JAX mice demonstrate significantly higher levels of lung fibrosis day 21 after bleomycin compared with CR mice (*P* < 0.05) measured by lung hydroxyproline content. (**D**) Gut microbiota depletion using broad-spectrum oral antibiotics results in significant protection for JAX mice from bleomycin-induced mortality (log rank *P* = 0.006). (**E**) Lung fibrosis is reduced in JAX mice treated with antibiotics (1 surviving mouse in control group) and CR mice treated with antibiotics. (**F**) Lung fibrosis is reduced in germ-free (GF) CR mice compared with conventional specific pathogen–free (SPF) CR mice. (**G** and **H**) Pulmonary interleukin-6 (IL-6) and leukemia inhibitory factor (LIF), a member of the IL-6 superfamily, are attenuated after bleomycin in CR mice compared with JAX. (**I**) Alveolar injury measured by IgM in bronchoalveolar lavage fluid day 7 after bleomycin is attenuated in CR mice compared with JAX mice. (**A**–**C** *n* = 20 per group, **D** and **E** *n* = 10 per group, **F** *n* = 5–6 per group, **G** and **H** *n* = 20 per group, **I** *n* = 10 per group.) Kaplan-Meier (KM) survival estimates and log rank test where applicable (**B** and **D**), unpaired *t* test or Mann-Whitney test (**C** and **F**–**I**), ANOVA (**E**), multiple *t* tests per row with correction for multiple comparison where applicable (**A**). *P < 0.05, **P < 0.01, ***P < 0.001.

We and others have previously shown that germ-free (GF) C57BL/6J (BL/6J — The Jackson Laboratory) mice are protected from lung fibrosis, supporting a causal role for microbiota. There are differences in gut microbiota between genetically identical mice sourced from different facilities that contributes to immunity (19). We hypothesized that BL/6J and Charles River Laboratories C57BL/6NCrl (BL/6NCrl) mice would exhibit differences in lung fibrosis outcomes as the result of vendor-derived gut microbiota heterogeneity promoting altered pulmonary immune responses. We induced lung fibrosis using bleomycin. Here, we show that BL/6NCrl mice were less susceptible to weight loss after bleomycin (*P* < 0.001) (Figure 1A). Weight loss by day 4 could be a key indicator of lung injury and associated with subsequent mortality. Relative percentage weight loss from baseline by day 4 after bleomycin treatment was associated with an increased hazard for mortality (1.55: 95% CI 1.27–1.90, *P* < 0.001) in a proportional hazards model. In surviving mice, weight loss by day 4 correlated robustly with lung fibrosis at day 21 (*r* = 0.58, *P* = 0.008), supporting acute weight loss as a surrogate of later stage lung fibrosis in this model (Supplemental Figure 1; supplemental material available online with this article; https://doi.org/10.1172/jci.insight.164572DS1). BL/6NCrl mice demonstrated increased survival compared with BL/6J mice after bleomycin (*P* < 0.0001) (Figure 1B). BL/6NCrl mice had less lung fibrosis after bleomycin compared with BL/6J mice measured by lung hydroxyproline content day 21 after bleomycin (*P* < 0.05) (Figure 1C). To verify that this observation was microbiota dependent, BL/6J and BL/6NCrl mice were treated with an established gut microbiota depletion approach using oral broad-spectrum antibiotics. BL/6J mice treated with antibiotics demonstrated improved survival compared with control untreated BL/6J mice after bleomycin (log rank *P* < 0.01) (Figure 1D) and less lung fibrosis overall than the surviving untreated BL/6J mouse (Figure 1E). BL/6NCrl mice treated with antibiotics exhibited reduced lung fibrosis compared with untreated BL/6NCrl mice after bleomycin. Finally, we and others have previously shown that GF C57BL/6J mice are protected from lung fibrosis. Here, we verify that the observation is also present in GF C57BL/6NCrl mice, where GF BL/6NCrl mice treated with bleomycin demonstrated attenuated lung fibrosis day 21 after bleomycin treatment compared with specific pathogen–free (SPF) BL/6NCrl mice (*P* < 0.05) (Figure 1F). We show that these findings correlated with the pulmonary interleukin-6 (IL-6) cytokine family. C57BL/6NCrl mice had lower levels of IL-6 and leukemia inhibitory factor (LIF), an IL-6 superfamily member, compared with C57BL/6J mice (Figure 1, G and H). In addition, we observed increased alveolar IgM in bronchoalveolar (BAL) fluid day 7 after bleomycin in BL/6J mice, supporting enhanced lung injury (Figure 1I). We concluded that there is heterogeneity in lung fibrosis outcomes in C57BL/6 mice derived from different vendor barrier facilities. Lung fibrosis is microbiota dependent in BL/6J and BL/6NCrl mice. Interestingly, in addition to microbiota-derived protection, BL/6NCrl mice may display host-inherent factors that contribute to this protection.

### C57BL/6 mice sourced from different vendors demonstrate distinct and heterogenous gut microbiota.

**Figure 2:**
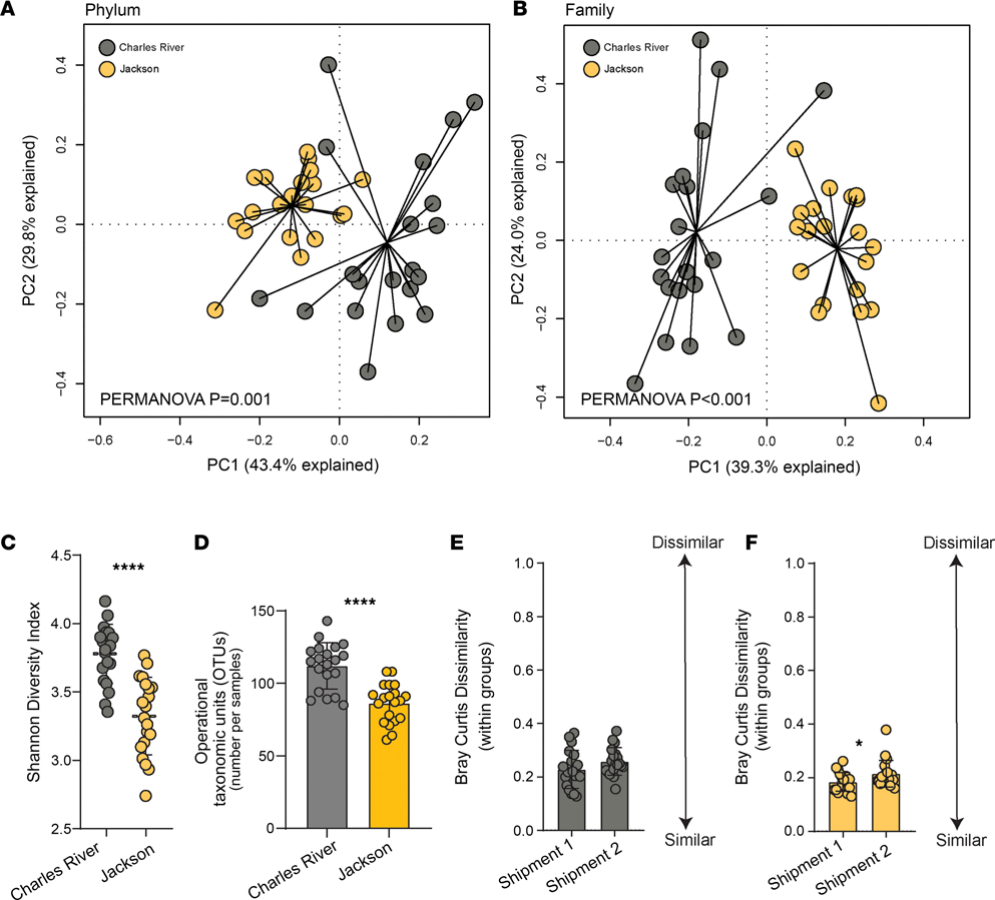
Substrain C57BL/6 mice from different barrier facilities display distinct and heterogenous gut microbial communities. Here, we characterized the gut microbiota of mice from JAX and CR laboratories on arrival to the University of Michigan. DNA was extracted from fecal pellets. 16S rRNA amplicon sequencing and shotgun metagenomics were used to analyze taxonomic composition and functional status of gut microbiota. (**A** and **B**) Principal component analysis (PCA) of 16S data with ordination demonstrates significant separation of microbial communities by C57BL/6 substrain at all taxonomic levels, including phylum (**A**) and family (**B**). (**C**) Shannon diversity indices are significantly higher in CR mice on arrival compared with JAX mice. (**D**) The number of unique operational taxonomic sequences identified in fecal samples is higher in CR mice compared with JAX mice on arrival. (**E** and **F**) Bray-Curtis dissimilarity scores of substrain mice from different facility shipments — where a score of 0 represents identical communities and a score of 1 represents no matching or shared taxa — JAX mice display significant within-shipment dissimilarity. (**A**–**F** *n* = 20 per group.) Permutational analysis of variance (PERMANOVA) where applicable (**A** and **B**), unpaired *t* test or Mann-Whitney test (**C**–**F**). *P < 0.05, ****P < 0.0001.

Given our hypothesis that gut microbiota heterogeneity is present in C57BL/6 mice derived from different facilities (21), we then tested this using 16S rRNA amplicon sequencing of DNA extracted from fecal pellets. On arrival, BL/6J and BL/6NCrl mice displayed grossly different gut microbial communities examined by principal component analysis (PCA) and ordination at multiple levels of taxonomic classification, including phylum (permutational multivariate analysis of variance [PERMANOVA] *P* = 0.001) (Figure 2A) and family (PERMANOVA *P* < 0.001) (Figure 2B). We examined gut microbial community dissimilarity using the Bray-Curtis dissimilarity score. BL/6J mice had dissimilar gut microbial communities from the BL/6NCrl (*P* < 0.0001) (Supplemental Figure 2). BL/6NCrl mice had greater α diversity measured by Shannon diversity indices (SDIs) (*P* < 0.001) (Figure 2C) compared with BL/6J mice and increased numbers of identifiable operational taxonomic units (OTUs) within gut communities (*P* < 0.001) (Figure 2D). We next used a generalized linear negative binomial model (GLM) to identify differentially abundant taxa between cohorts. This model was statistically significant (*P* < 0.001) (Supplemental Table 1). Taxa at increased abundance in BL/6NCrl mice compared with BL/6J mice included Clostridiales unclassified (*P* = 0.001), Lactobacillaceae (*P* = 0.001), and Deferribacteraceae (*P* = 0.004). We report several taxa at increased abundance in BL/6J mice compared with BL/6NCrl, including Erysipelotrichaceae (*P* = 0.001), Verrucomicrobiaceae (*P* = 0.001), Ruminococcaceae (*P* = 0.02), Bifidobacteriaceae (*P* = 0.002) and Anaeroplasmataceae (*P* = 0.003). Analysis of different vendor shipments demonstrates differences in taxonomic composition of gut microbiota. BL/6NCrl mice did not demonstrate significant within-shipment dissimilarity (*P* > 0.05) (Figure 2E). However, BL/6J mice demonstrated significant within-shipment dissimilarity (*P* < 0.05) (Figure 2F). Thus, gut microbiota from substrain mice are altered at all levels of taxonomic classification, vary with facility barrier rooms, and contain notable keystone taxa with plausible biological implications.

### Gut microbiota are altered during acute lung injury and lung fibrosis, displaying distinct patterns of change.

**Figure 3:**
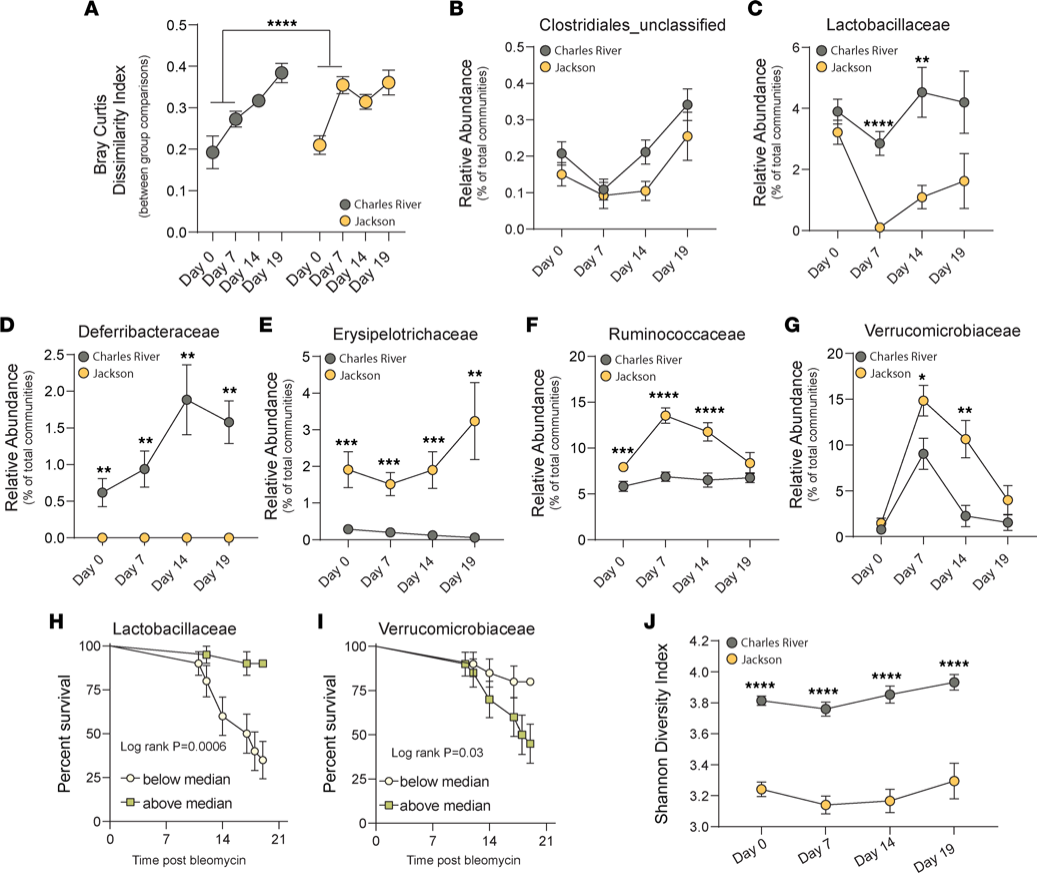
Substrain C57BL/6 mice demonstrate varied patterns of dysbiosis that associate with mortality and lung fibrosis. Here, we analyzed the response of gut microbiota during stages of acute lung injury (day 7 after bleomycin), transition of lung fibrosis (day 14), and advanced lung fibrosis (study end) using 16S rRNA amplicon sequencing in CR and JAX substrain mice. (**A**) Bray-Curtis dissimilarity scores show a significant increase in dissimilarity from baseline (day 0) to day 7 in JAX mice, demonstrating an increased susceptibility to gut dysbiosis. (**B**) No significant difference between groups in abundance of Clostridiales over time. (**C**) Significant decrease in the abundance of potentially antiinflammatory Lactobacillaceae by day 7 after bleomycin with persistently higher levels of Lactobacillaceae in CR mice compared with JAX mice over time. (**D**) Significant increase in abundance of Deferribacteraceae in CR mice after bleomycin correlating with survival; this taxon was undetectable in JAX mice. (**E**–**G**) Significant increases in Erysipelotrichaceae and Ruminococcaceae over time in JAX mice after bleomycin compared with CR mice, with significant increase in Verrucomicrobiaceae in both CR and JAX mice; however, the effect size is significantly greater in JAX mice, and abundance remains higher in JAX mice over time. (**H**) The relative abundance of gut Lactobacillaceae at baseline is associated with a reduced risk of mortality; mice with a relative abundance greater than 1.42% (median) were protected from mortality (log rank *P* < 0.001). (**I**) An increased relative abundance of gut Verrucomicrobiaceae at baseline was associated with an increased risk of mortality, mice with a relative abundance greater than 5.55% of total communities were at significantly greater risk of mortality (log rank *P* = 0.03). (**J**) CR mice demonstrated significantly higher α diversity measured by Shannon diversity indices throughout the experiment. (**A**–**J** *n* = 20 per group.) Unpaired *t* test or Mann-Whitney test where applicable (**A**), multiple *t* test per row with multiple comparison corrections where applicable (**B**–**G** and **J**), KM survival estimates and log rank test (**H** and **I**). *P < 0.05, **P < 0.01, ***P < 0.001, ****P < 0.0001.

We next tested whether survival in BL/6NCrl mice would correlate with distinct changes in gut microbial communities that were substrain dependent. A key finding is that BL/6J mice demonstrated a marked change in Bray-Curtis dissimilarity between the time of bleomycin treatment (day 0) and the time of approximate peak lung injury (day 7), the effect size of which was not seen in BL/6NCrl mice (*P* < 0.0001) (Figure 3A). BL/6NCrl mice exhibited incremental increases in dissimilarity over time (Figure 3A). We next examined the changes in taxa that were noted to be differentially abundant in BL/6NCrl and BL/6J mice at baseline. There were no changes noted in the abundance of Clostridiales in both cohorts of mice after bleomycin (Figure 3B). There was a decrease in the abundance of Lactobacillaceae in BL/6J mice after bleomycin, which did not occur in BL/6NCrl mice (*P* < 0.0001) (Figure 3C). BL/6NCrl mice demonstrated relatively stable gut Lactobacillaceae community abundance. There were also notable and significant increases in Deferribacteraceae in BL/6NCrl mice after bleomycin (*P* < 0.01) (Figure 3D) correlating with survival. This taxon was undetectable in BL/6J mice. In BL/6J mice, there were increases in Erysipelotrichaceae (*P* < 0.001) (Figure 3E), Ruminococcaceae (*P* < 0.001) (Figure 3F), and Verrucomicrobiaceae (*P* < 0.01) (Figure 3G) after bleomycin compared with BL/6NCrl mice. The relative abundance of Lactobacillaceae at baseline in this experiment was associated with protection against mortality (HR 0.56 95%, CI 0.37–0.85, *P* = 0.006). When stratified by above- or below-median abundance (1.42%), mice with an abundance of Lactobacillaceae above the median had a significantly reduced risk of mortality compared with mice with a relative abundance below the median (*P* < 0.001) (Figure 3H). An increased abundance of Verrucomicrobiaceae was associated with an increased risk of mortality at baseline (HR 1.21, 95% CI 1.06–1.38, *P* = 0.004) Again when stratified by median abundance (5.55%), an increased abundance greater than the median was associated with an increased risk of mortality (*P* = 0.03) (Figure 3I). Finally, we examined α diversity of gut microbial communities in BL/6J and BL/6NCrl mice over time after bleomycin. BL/6NCrl mice demonstrated greater SDI compared with BL/6J mice during the acute and chronic stages of the lung fibrosis model, which correlated with protection in BL/6NCrl mice (Figure 3J). Overall, we demonstrate that during the acute lung injury phase of the model, there are changes in community composition, with greater community stability and an increased abundance of potentially antiinflammatory and antifibrotic taxa in BL/6NCrl mice compared with BL/6J mice correlating with protection against fibrosis.

### Substrain C57BL/6 mice harbor distinct immunological profiles that correlate with gut microbiota in lung fibrosis.

**Figure 4:**
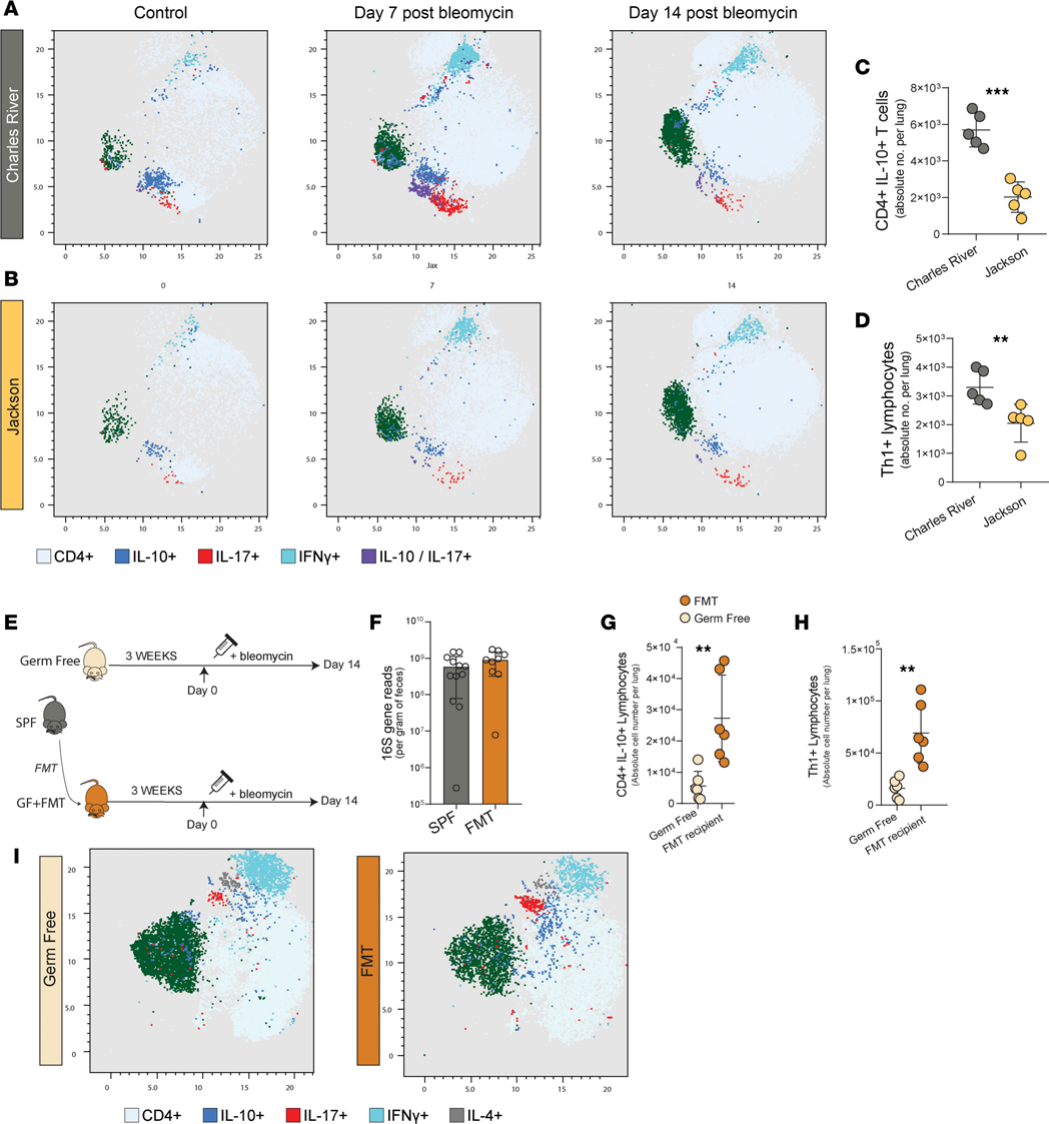
Pulmonary immunity is regulated by gut microbiota. Lung fibrosis was induced with bleomycin and C57BL/6Cr and C57BL/6J mice underwent collagenase digestion of a lung lobe to isolate pulmonary leucocytes. Multicolor flow cytometry was used to characterize cells. Samples were concatenated in FlowJo v10.5 and pregated as CD45^+^. (**A** and **B**) UMAP of leucocytes isolated from whole-lung collagenase digestion in CR and JAX mice in control mice, day 7 after bleomycin and day 14 after bleomycin. (**C**) In control mice there are increased CD4^+^IL-10^+^ T cells and (**D**) Th1^+^ lymphocytes at baseline in CR mice compared with JAX correlating with varied gut microbiota. (**E**) Study design for fecal microbiota transplantation. Fecal microbiota transplant (FMT) was achieved by oral gavage of slurry followed by a 3-week period, fibrosis was induced with bleomycin in C57BL/6Cr mice, and outcomes were examined day 14 after bleomycin. (**F**) No differences were noted in sample bacterial density from fecal pellets in FMT and SPF mice. (**G**). Higher levels of CD4^+^IL-10^+^ T cells in FMT recipients and (**H**) higher levels of Th1^+^ T cells in FMT recipients day 14 after bleomycin. (**I**) UMAP of pulmonary leucocytes in GF mice and FMT recipients. In all UMAPs, green represents regulatory T cells. (**A**–**D** *n* = 5 per group, **F**–**I** *n* = 6–8 per group.) Unpaired *t* test, Mann-Whitney test (**C**, **D**, **G**, and **H**) where applicable. *P < 0.05, **P < 0.01, ***P < 0.001, ****P < 0.0001.

Gut microbiota interactions with the host are known regulators of systemic cellular immunity. Here, we examined the pulmonary cellular immune response after bleomycin in BL/6J and BL/6NCrl mice. BL/6NCrl and BL/6J mice went untreated or received bleomycin and underwent collagenase digestion of whole lung to isolate leucocytes at varied time points after bleomycin. Leucocytes were stained with multiple antibodies to identify both innate and adaptive immune cell types, and uniform manifold approximation and projection (UMAP) was used to visualize differences in BL/6NCrl and BL/6J mice at key time points in the lung fibrosis model (Figure 4, A and B). We used an ensemble machine learning random forest technique to identify differences in the frequency of lung immune cell types in untreated, nonfibrotic BL/6NCrl and BL/6J mice (Supplemental Figure 3). We report increases in CD4^+^IL-10^+^ T cells at baseline, in untreated, nonfibrotic mice (*P* < 0.001) (Figure 4C) as well as increases in Th1 lymphocytes (*P* < 0.01) (Figure 4D). We report at day 7 after bleomycin a persistent increase in CD4^+^IL-10^+^ T cells within the lung microenvironment of BL/6NCrl mice compared with BL/6J mice (*P* < 0.01) (Supplemental Figure 4). To study the potential causal impact of gut microbiota on the pulmonary immune cell response in lung fibrosis, we next used GF mice — experimental mice devoid of microbiota — and fecal microbiota transplantation (FMT). Here, FMT was achieved in GF (C57BL/6NCrl) recipients by oral gavage of fecal slurry from SPF controls (Figure 4E). Three weeks after FMT, gut microbial communities were detectable in FMT recipient mice, with similar levels of bacterial density per sample (Figure 4F) and with significantly varied community composition between FMT and BL/6NCrl donor mice measured by PCA and ordination (PERMANOVA *P* = 0.001) (Supplemental Figure 5). The oral gavage did not fully recapitulate microbial communities in FMT recipient mice from donor BL/6NCrl mice but established an identifiable community. FMT recipients and GF mice were treated with bleomycin, and pulmonary immune cell responses were analyzed using multicolor flow cytometry after collagenase digestion of lung 14 days after treatment. We noted key changes in the adaptive immune response with increased numbers of CD4^+^IL-10^+^ lymphocytes (*P* < 0.01) (Figure 4G) and Th1^+^ lymphocytes (*P* < 0.01) (Figure 4H) in FMT recipient mice compared with GF mice. Immune cell types reported to vary and correlate with gut microbiota in BL/6J and BL/6NCrl mice were displayed by UMAP (CD4^+^IL-10^+^ T cells, Th1^+^ lymphocytes) (Figure 4I) and were quantitatively dependent on the presence of microbiota. We concluded that a causal association exists between microbiota and pulmonary cellular immune responses in lung injury and fibrosis.

### Horizontal gut microbiota transmission through cohousing attenuates lung fibrosis.

**Figure 5:**
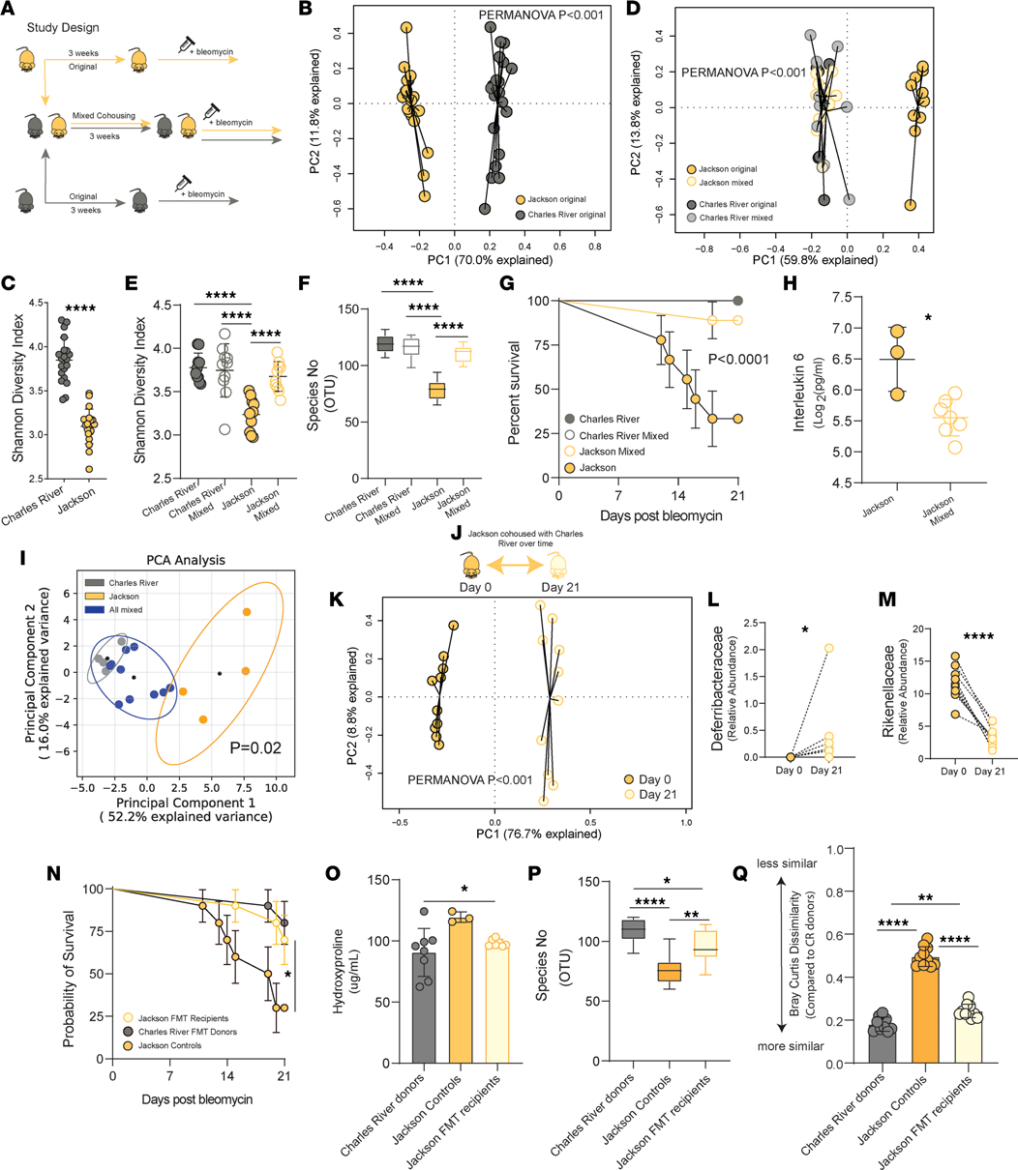
Horizontal transmission of gut microbiota can modify experimental outcomes in lung fibrosis models. 16S rRNA amplicon sequencing of feces and lung fibrosis outcomes after bleomycin treatment. (**A**) C57BL/6J and C57BL/6Cr mice were randomly assigned 1:1 to shared cages for mixing, while the remaining original cohort of C57BL/6J and C57BL/6Cr mice were left in the same cages for 3 weeks. (**B**) Gut microbiota demonstrates distinct community composition by PCA and ordination at baseline. (**C**) Shannon diversity is significantly greater in CR mice at baseline. (**D**) PCA and ordination demonstrate a coalescence of gut microbial communities in shared mixed cages, with C57BL/J original cages remaining significantly altered. (**E**) Cohousing increases diversity in BL/6J mice housed. (**F**) Increased operational taxonomic units (OTUs) identified in mixed C57BL/6J mice. (**G**) Survival after bleomycin, mixed BL/6J mice have improved survival. (**H**) Whole-lung IL-6 is reduced in mixed BL/6J mice. (**I**) PCA of GSEA enriched immunological signatures in original BL/6J and BL/6Cr mice compared with all mixed mice (permutational multivariate analysis of variance [PERMANOVA] *P* = 0.02 for BL/6J compared with BL/6Cr). (**J**) Study schematic. (**K**) PCA and ordination of BL/6J mice from day 0 of cohousing with BL/6Cr mice to day 21. (**L** and **M**) Changes in taxa over time in BL/6J mice mixed with BL/6Cr mice. (**N**) Reduced mortality in BL/6J FMT recipients from BL/6NCrl donors compared with BL/6J non-FMT controls, log rank *P* = 0.04 BL/6J recipients (30% mortality) versus BL/6J control (70% mortality). (**O**) Reduced lung collagen content in BL/6J recipients. (**P**) Significant horizontal transfer of OTUs by FMT. (**Q**) Increased similarity in BL/6J FMT recipients compared with BL/6NCrl donors on FMT to BL/6J from NCrl donors measured by Bray-Curtis dissimilarity. (**A**–**Q** *n* = 10 per group.) Box plots show the interquartile range (box), median (line), and minimum and maximum (whiskers). PERMANOVA (**B**, **D**, and **I**), unpaired *t* test or Mann-Whitney test (**C**, **H**, **L**, and **M**), ANOVA (**E**, **F**, and **O**–**Q**), log rank *t* test (**G** and **K**). *P < 0.05, **P < 0.01, ****P < 0.0001.

To evaluate further the causal contribution of gut microbiota and given that GF mice are less susceptible to bleomycin-induced lung fibrosis, we leveraged our previous observations whereby 3 weeks of cohousing experimental mice will promote the horizontal transmission of microbiota and increase community similarity within cohoused cohorts (22). We cohoused by mixing BL/6J and BL/6NCrl mice for 3 weeks or left BL/6J and BL/6NCrl mice in original cohorts and induced lung fibrosis in all (Figure 5A). At baseline, we noted again that community composition was significantly different between BL/6J and BL/6NCrl cohorts measured by PCA and ordination (PERMANOVA *P* < 0.001) at the family level of taxonomic classification (Figure 5B). We examined Bray-Curtis dissimilarity scores between cohorts. When measuring dissimilarity between BL/6NCrl and BL/6J mice, we report, as expected, increased dissimilarity in BL/6J mice compared with BL/6NCrl (*P* < 0.0001) (Supplemental Figure 6), which supports our observation by PCA and validates our previous findings.

We then examined diversity measured by SDI and report significantly greater diversity in gut microbial communities of mice sourced from BL/6NCrl compared with BL/6J (*P* < 0.0001) (Figure 5C). We used a GLM to identify differentially abundant taxa between cohorts. The model was significant (*P* < 0.001) (Supplemental Table 2) and identified 18 taxa after controlling conservatively for multiple comparisons, including Bifidobacteriaceae (*P* = 0.001), Erysipelotrichaceae (*P* = 0.001), Deferribacteraceae (*P* = 0.001), Lactobacillaceae (*P* = 0.001), Verrucomicrobiaceae (*P* = 0.001), Anaeroplasmataceae (*P* = 0.003), and Clostridiales (*P* = 0.004) (Supplemental Table 2), validating our previous findings. After 3 weeks of cohousing, changes in community composition were noted, with the greatest change seen in BL/6J mice mixed with BL/6NCrl mice. PCA and ordination demonstrated clustering of mixed BL/6J, mixed BL/6NCrl, and original BL/6NCrl mice (Figure 5D). There were no significant differences in gut microbial community composition between mixed BL/6J mice and mixed BL/6NCrl mice (PERMANOVA *P* > 0.05) or nonmixed BL/6NCrl mice (PERMANOVA *P* > 0.05) after 21 days. We report a significant difference between original BL/6J mice and mixed BL/6J mice at 21 days (PERMANOVA *P* < 0.001) (Figure 5D). We examined dissimilarity scores between communities in each cohort after 21 days. Compared with the original BL/6J cohort, we report reduced dissimilarity in the BL/6J mixed cohort (becoming more similar to BL/6NCrl) (*P* < 0.0001) but increased dissimilarity in the BL/6J original cohort compared with BL/6NCrl (*P* < 0.0001) (Supplemental Figure 7), complementing our observations by PCA. We then examined diversity by SDI and report that gut microbial communities of BL/6J mixed mice exhibited increased community diversity after 21 days (*P* < 0.001) compared with original BL/6J mice (Figure 5E). We report an increase in identifiable OTUs in cohoused BL/6J mice compared with original BL/6J mice (*P* < 0.0001) (Figure 5F). In brief, mixing of BL/6J mice with BL/6NCrl mice resulted in the horizontal transmission of gut microbiota across biological gradients, with mixed BL/6J mice gaining taxa, increased α diversity, and increased similarity to BL/6NCrl mice.

We then treated experimental mice with bleomycin to induce lung fibrosis. The original BL/6NCrl mice were protected from mortality compared with original BL/6J mice (0% vs. 66% mortality, log rank *P* < 0.0001) (Figure 5G). However, BL/6J mice mixed with BL/6NCrl mice demonstrated significantly reduced mortality (11% vs. 66%, log rank *P* = 0.01) compared with original BL/6J mice. In surviving mice, we found no significant difference in lung collagen content in BL/6NCrl mice and BL/6J mice, with a tend toward reduced collagen content in BL/6J mixed mice compared with BL/6J mice (Supplemental Figure 8). However, the assessment was limited because of the high mortality in the BL/6J original cohort. We analyzed concentrations of IL-6 in the lungs of BL/6J mice and BL/6J mice mixed with BL/6NCrl mice. The BL/6J mice mixed with BL/6NCrl mice demonstrated a significant reduction in IL-6 (*P* < 0.05) (Figure 5H). We then analyzed the pulmonary transcriptomic signature of BL/6J mice mixed with BL/6NCrl mice. Using gene set enrichment analysis (GSEA) and the Molecular Signatures Database (MSigDB) immune signatures database, we isolated 138 enriched immune genes and compared them using PCA (Figure 5I) (Supplemental Table 3). There were significant differences in immune transcripts between BL/6J and BL/6NCrl mice (PERMANOVA *P* = 0.02). When we examined all mixed mice, the enriched gene set was not significantly different from BL/6NCrl mice (PERMANOVA *P* = 0.38), supporting our hypothesis that the transmission of gut microbiota altered the pulmonary immune microenvironment. We next identified candidate taxa that were successfully transmitted from BL/6NCrl mice to BL/6J mixed mice by examining differences between BL/6J original and BL/6J mixed mice (Figure 5J). A GLM identified changes between BL/6J mixed and BL/6J original mice with loss or gain of multiple gut taxa after 21 days (*P* < 0.001) (Supplemental Table 4), including changes in Erysipelotrichaceae, Anaeroplasmataceae, and Deferribacteraceae. In mixed BL/6J mice we report the gain and loss of several candidate taxa over time (PERMANOVA *P* < 0.001) (Figure 5K). We report a gain/transmission of Deferribacteraceae (*P* < 0.05) (Figure 5L), in addition to Prevotellaceae (*P* < 0.01), Bacteroidales unclassified (*P* < 0.01), Bacteroidetes unclassified (*P* < 0.01), and Desulfovibrionales unclassified (*P* < 0.05), in cohoused BL/6J mice over time in addition to others (Supplemental Figure 9). We report a reduction in the relative abundance of gut Rikenellaceae (*P* < 0.0001) (Figure 5M), Bifidobacteriaceae (*P* < 0.01), Erysipelotrichaceae (*P* < 0.01), Bacteroidaceae (*P* < 0.0001), and Anaeroplasmataceae (*P* < 0.05) in cohoused BL/6J mice over 21 days (Supplemental Figure 9). Finally, we examined changes over time in taxa after bleomycin in both substrains. We report a gain of Lactobacillaceae in BL/6NCrl mice, an increase in Rikenellaceae in BL/6J original mice, an increase in Bacteroidaceae in BL/6J mice over time after bleomycin, and an increase in Verrucomicrobiaceae in both cohorts (Supplemental Figure 10). Several taxa remained undetectable in BL/6J original mice over time but were abundant in all other groups, including Deferribacteraceae and Desulfovibrionales (Supplemental Figure 10). To evaluate this hypothesis further, we performed FMT in SPF BL/6J mice, whereby the BL/6J mice either received an FMT from BL/6NCrl donors or did not. BL/6J FMT recipients had improved survival compared with BL/6J controls (log rank *P* = 0.04) (Figure 5N) and lung fibrosis compared with BL/6J controls (*P* < 0.05) (Figure 5O). FMT increased the number of detectable OTUs within the feces of BL/6J FMT recipients (Figure 5P) and increased the similarity between BL/6J FMT recipients and BL/6NCrl donors, verifying the horizontal transfer of microbiota (Figure 5Q). The data support a causal association that is readily modifiable through transmission of gut microbiota. The model circumvents the abnormal baseline immunity seen in complex GF murine models. However, a comparison across models supports our opinion that cohousing is more effective at horizontal transfer than FMT.

### Shotgun metagenomics of gut microbiota identifies keystone species in substrain mice.

**Figure 6:**
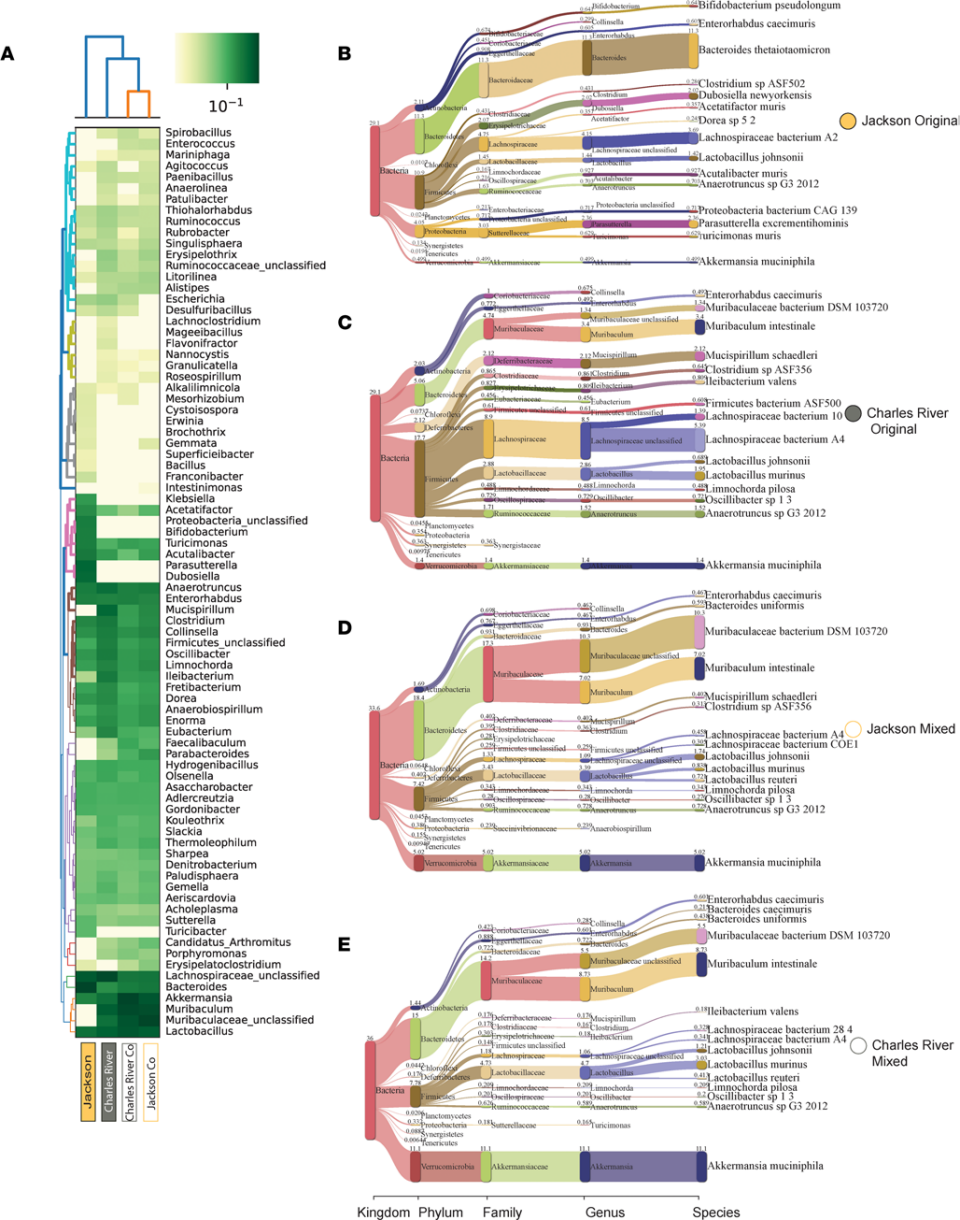
Advanced shotgun metagenomic characterization of gut microbial communities in C57BL/6 substrain mice. Here, we used advanced genomic sequencing and MetaPhlAn to characterize distinct taxonomic composition of gut microbial communities. Analysis was performed on DNA extracted from fecal samples in representative experimental mice from each group. Between 29.1% and 36% of reads were mappable using this tool. (**A**) Heatmap visualization and hierarchical clustering at the genus level. (**B**–**E**) Sankey plots of the 15 most abundant species in each representative sample.

We have identified heterogeneity of gut microbial communities between different vendor-sourced mice on the same genetic background and characterized this at the family taxonomic level using 16S amplicon sequencing data. To further understand the changes occurring in microbial communities, we used advanced shotgun metagenomics of DNA extracted from fecal samples to characterize in detail the taxonomic changes at the genus and species level. These samples were analyzed after 3 weeks of cohousing and before bleomycin treatment. A heatmap was generated from these data, and this clearly demonstrated separation at the genus level, with keystone species noted. *Mucispirillum*, *Muribaculum*, and *Muribaculaceae* were abundant genera detectable in gut communities of BL/6NCrl mice but not found in BL/6J mice (Figure 6A). In BL/6J mice we identified several taxa that were not detectable in BL/6NCrl mice, including *Parasutterella*, *Dubosiella*, *Proteobacteria bacterium*, *Klebsiella*, and *Bifidobacterium*. A heatmap of species level data is included in the supplement (Supplemental Figure 11). We next catalogued the taxonomic differences at the species level visualized using Sankey plots (ranked by top 10 most abundant species). The differences are clear with mixed cohousing where BL/6J mice incur the loss of *Bacteroides thetaiotaomicron* and the gain of *Muribaculum intestinale*, *Muribaculaceae bacterium*, and *Mucispirillum schaedleri* among others establishing a community similar to BL/6NCrl mice (Figure 6, B–E). These species represent several candidate taxa that may be involved in the pathogenesis of lung injury and lung fibrosis.

### The selective contributions of lung and gut microbiota in the regulation of lung injury and fibrosis.

**Figure 7:**
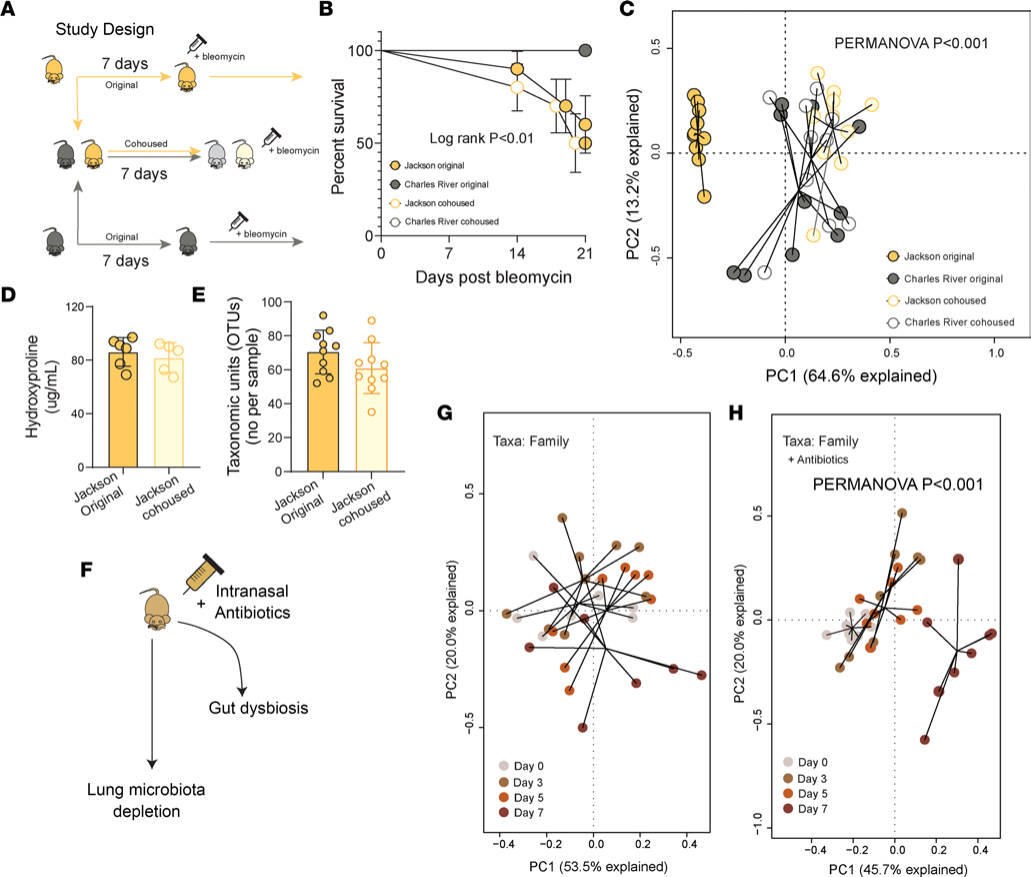
Short-term cohousing is less effective and does not provide protection in preclinical models of bleomycin-induced lung fibrosis. (**A**) Study design: C57BL/6Cr mice are cohoused with C57BL/6J mice for 7 days and then treated with bleomycin to induce lung fibrosis. (**B**) Kaplan-Meier estimates of survival show that Jackson mice cohoused with Charles River mice for 7 days do not demonstrate protection against lung fibrosis and have similar increased mortality compared with Jackson mice that were not cohoused with Charles River. (**C**) PCA and ordination of 16S data after 7 days of cohousing. Community composition has shifted, but C57BL/6J cohoused mice remain significantly different by PERMANOVA from others. (**D**) Similar levels of lung collagen content in Jackson mice and Jackson mice cohoused with Charles River mice. (**E**) Equal number of identifiable taxa in both Jackson cohorts. (**F**) Study design: to understand the potential impact of intranasal antibiotics on gut microbiota, we gave intranasal antibiotics to C57BL/6J mice and then examined gut microbiota composition by 16S rRNA amplicon sequencing over 7 days. (**G**) Control mice receiving intranasal sterile saline did not demonstrate significant changes in gut microbial community composition over time (PERMANOVA *P* > 0.05). (**H**) PCA and ordination of 16S data from mice receiving intranasal antibiotics demonstrate a significant separation of community composition by day 7 (PERMANOVA *P* < 0.001). (*n* = 8–10 per group.) PERMANOVA (**C**, **G**, and **H**), log rank test (**B**), unpaired *t* test or Mann-Whitney test (**D** and **E**) where applicable. *P < 0.05, **P < 0.01, ****P < 0.0001.

In preclinical modeling, we and others have shown that the lung microbiota are altered in the bleomycin lung fibrosis model and host and commensal lung microbiota interactions promote pathogenesis (13). Others have proposed a negligible role for gut microbiota in the bleomycin model as orally administered antibiotics were not associated with protection from fibrosis, and intranasally administered antibiotics resulted in attenuated lung fibrosis (9). Here we sought to improve our understanding of contributions of lung and gut microbiota in our models. Our initial studies demonstrated that in our models oral antibiotics protected against lung fibrosis. Previous work has demonstrated that the murine lung microbiome of BL/6J and BL/6NCrl increases in similarity within 24 hours, reaching maximal compositional similarity at 7 days. In the same experiments corresponding gut microbiota remained distinct at 7 days. We cohoused BL/6J and BL/6NCrl mice for 7 days, ensuring lung microbiota similarity (while gut microbial communities remain distinct), then treated with bleomycin to induce lung fibrosis (Figure 7A). BL/6J mice and BL/6J mice cohoused with BL/6NCrl mice demonstrated similar increased mortality compared to cohorts of BL/6NCrl mice (log rank *P* = 0.6 for BL/6J compared to BL/6J cohoused) (Figure 7B). We examined gut microbial communities by 16S rRNA amplicon sequencing of feces after 7 days of cohousing between BL/6J and BL/6NCrl mice before administration of bleomycin. On day 7 there were notable shifts in gut microbial communities in cohoused BL/6J and BL/6NCrl mice as analyzed by PCA and visualized by ordination (Figure 7C). BL/6J original mice remained clustered separately from all groups; however, BL/6J cohoused and BL/6NCrl cohoused mice demonstrated different taxonomic composition (PERMANOVA *P* = 0.05), while BL/6NCrl original mice exhibited different community composition compared with BL/6J cohoused (PERMANOVA *P* = 0008). There was no difference in lung fibrosis as measured by total lung collagen in both BL/6J and cohoused BL/6J mice (Figure 7D). No signficant increase in gut OTUs was noted on short-term cohousing (Figure 7E). These data support our hypothesis that the protective effect in our cohousing model is largely dependent on the horizontal transmission of gut microbiota over time. As intranasal antibiotics are associated with protection from bleomycin-induced lung fibrosis, we next determined whether intranasal antibiotics are associated with altered gut microbial community composition. We gave intranasal antibiotics as previously reported to 2 cohorts of mice and then determined gut community composition by 16S rRNA amplicon sequencing of feces over 7 days (Figure 7F). Gut microbial communities were studied using PCA and ordination. Control experimental mice received sterile saline intranasally, and no changes were seen in the composition of gut microbiota over time (PERMANOVA *P* > 0.05) (Figure 7G). However, in mice receiving intranasal antibiotics, we report a significant change in gut microbial communities over time (PERMANOVA *P* < 0.001) (Figure 7H). There was no difference in bacterial burden as measured by 16S gene reads per gram of feces (data not shown). In summary, the transmission of lung microbiota over 7 days was not associated with protection from lung fibrosis in these models. Intranasal antibiotics alter the lung microbiome but also induce gut dysbiosis.

## Discussion

The gut microbiome has a substantial impact on human health (1). Recent work has supported a causal relationship between microbiota and immune cell dynamics in humans, plausibly linking gut dysbiosis with inflammatory and immune-mediated disease (2). Lung fibrosis is generally regarded as the sequela of acute and/or subclinical lung injury mediated by key features of the immune system (12). In this work, we establish a role for gut microbiota in regulating lung injury and lung fibrosis. We show that mortality in preclinical models is microbiota dependent, that vendor-sourced substrain mice have distinct microbiota that regulates the lung immune response, and that microbiota can be transmitted horizontally to attenuate mortality in these models.

Gut microbiota are master regulators of immunity, a process achieved through altered innate immune cells, inflammatory cytokine responses, and reprogramming of adaptive immunity, likely in addition to other poorly understood mechanisms (24, 25). Through these actions, the gut microbiome can exert immune-mediated effects on remote organs, including the lung. The absence of microbiota in GF mice or microbiota depletion with antibiotics in models of pulmonary infection results in altered susceptibility to mortality (26, 27). FMT can rescue host defense and restore survival in these infection models (26, 28). We and others have shown that GF mice are protected from pulmonary fibrosis induced by a sterile lung injury (9, 13). Here, we show that taxonomic composition and α diversity, key features of the gut microbiome, correlate with survival in lung fibrosis. We show that these related effects are transferable through cohousing. These observations have important implications for the scientific rigor and reproducibility of future work in biological models of lung fibrosis. Work is now needed to study this consortium of protective taxa and understand how they colonize a susceptible host environment to alter cell immunity and protect against fibrosis. Previous work has highlighted the predominance of vertical transmission of microbiota in mammals through maternal ancestry (21). However, studies in humans now report that a low portion of microbiota are heritable and that horizontal transmission through cohabitation contributes substantially to microbiome composition (3).

The gut microbiome has proven associations with T cell immunity and reprogramming of adaptive immunity. Commensal microbiota in the gut environment can generate products that induce IL-10–producing regulatory T cells, with subsequent amelioration of experimental gut injury (29, 30). We and others have previously shown that peripheral blood proteomic signatures in patients with idiopathic pulmonary fibrosis (IPF) support dysregulated T cell immunity (31). It has been hypothesized that certain microbiota exposures induce T cell differentiation in gut-associated lymphoid tissue whereby cells may then migrate to the lung in response to inflammatory stimuli (32). Other studies have shown that vendor-specific microbiota at baseline correlate with levels of FoxP3^+^ T cells (33). In IPF, T cells have been shown to be protective in lung fibrosis in preclinical models, and regulatory T cells are globally impaired in patients with IPF (34, 35). Key yet poorly understood relationships exist between gut microbiota and T cell immunity in lung fibrosis that require further study.

Lung microbiota are altered in models of lung fibrosis, and this lung dysbiosis precedes peak lung injury (13). In patients with IPF, increased respiratory tract bacterial burden has been consistently associated with an increased risk of death and disease progression (13, 14, 36). Studies have previously sought to exclude a role for gut microbiota in lung fibrosis using oral antibiotics to deplete gut microbiota, demonstrating a negligible effect on lung fibrosis. However, the myeloid cell pool is regulated by circulating gut microbiota products (8, 37), gut microbiota are reported to regulate the accumulation of pulmonary leucocytes in response to pulmonary infection (7), and T cell immunity is subject to reprogramming on exposure to gut microbiota–derived products/metabolites (5, 38, 39). Here, we show that oral antibiotics can protect against lung fibrosis in preclinical models and that on horizontal transmission the effect of protective gut microbiota predominates once stable communities have been established. This work suggests that gut microbiota regulate immune cell dynamics in these models of lung injury, while lung microbiota largely associate with local alveolar cytokine responses, supporting contributions from both mucosal communities to disease pathogenesis.

Given ancestry in substrain BL/6J and BL/6NCrl mice, over time genetic “drift” has occurred, which may contribute to lung injury and fibrosis phenotypes in these C57BL/6 substrains (40). There are significant differences in gene expression in C57BL/6 substrain mice because of “genetic drift,” with studies demonstrating changes between strains in cardiovascular responses, responses to intermittent hypoxia, skeletal and muscle responses to high-fat diet, and susceptibility to influenza A infection in addition to others (41–45). However, this ancestry has resulted in the evolution of distinct and heterogenous gut microbiota with minimal shared taxa between substrains. Given the regulatory role gut microbiota occupy in the immune system and the ability to protect against mortality through successful transfer of gut microbiota by cohousing, our work supports a large contributory role for gut microbiota in these models of lung fibrosis. We hypothesize that the horizontal transfer from BL/NCrl mice to BL/6J mice is the result of significantly higher diversity and overall identifiable taxa in the BL/6NCrl mice. Given coprophagia, it is possible that some of this effect was the result of behavioral changes in mice on cohousing. Importantly, BL/6NCrl mice were still protected from mortality on gut microbiota depletion with antibiotics, suggesting that the contribution of gut microbiota was partial, while BL/6J mice had survival restored in this setting, supporting a more complete contribution to phenotype.

There are notable limitations to our study. We identify overall changes in T cell immunity, innate immunity, and pulmonary IL-6 cytokine family levels and provide supporting correlative evidence with gut microbiota. While the exact mechanisms of protection are multifactorial, further work is required to understand specific cell, cytokine, and microbiota product interactions. Diet is an important modifier of gut microbial communities; while both BL/6J and BL/6NCrl mice are fed different diets at the vendor barrier facility, on arrival to our facility they are fed the same standard diet. Recent work has substantiated our findings, demonstrating that within institutional barrier facilities using FMT, identical mice demonstrate varied lung fibrosis responses that are gut microbiota dependent and potentially mediated through dysregulated IL-6 signaling and augmented neutrophil recruitment to the injured lung (10, 11).

In summary, we have determined that lung fibrosis is microbiota dependent, identified variation in the gut microbiome of vendor-sourced C57BL/6 substrain mice, and correlated microbiota with different lung fibrosis phenotypes. We also show that microbiome-mediated effects on lung fibrosis are transferable to a susceptible host. Given genetic drift among C57BL/6 substrains over time, further work is required to delineate and clarify contributions of microbiota to host phenotype and genome-microbiome interactions. Our work promotes a changing paradigm in lung fibrosis and establishes rationale for further mechanistic work and patient-centered studies.

## Methods

### Experimental mice.

Six- to 8-week-old mice were sourced from commercial vendors (The Jackson Laboratory [C57BL/6J] or Charles River Laboratory [C57BL/6NCrl]) and transported to the University of Michigan laboratory animal care facility. Mice were housed under SPF conditions and received water and standard chow ad libitum. Diet provided was standard university laboratory rodent chow (Purina lab diet 5LOD) and consistent across all experimental groups and all experiments described herein. GF mice were fed autoclaved LabDiet 5013 and autoclaved water. Cage bedding was changed according to institutional timelines and protocols. Mice in all studies were housed under a 12-hour light/12-hour dark cycle at a constant temperature of 70 ± 2°C with a relative humidity of 30%–50%. GF mice on a C57BL/6NCrl background were bred and maintained by the Unit for Laboratory Animal Medicine’s Germ-Free Mouse Facility at the University of Michigan and were housed in flexible-film isolators or in positive-pressure individually ventilated cages. All handling of GF mice was done with strict aseptic technique. Feces of GF mice were assessed for GF status on a regular basis using Gram stain and culture (aerobic and anaerobic). Cohousing experiments involved the transfer of female mice after ear tagging in a 1:1 randomization from the original cohort to labeled cages. Intranasal antibiotics were given by intranasal injection of a volume of 50 μL of double-distilled water containing ampicillin (1 mg/mL), vancomycin (0.5 mg/mL), metronidazole (1 mg/mL), and neomycin (1 mg/mL) given once a week or sterile saline. Oral antibiotics were administered in drinking water at the same concentration. Data in experiments with SPF mice represent 2 independent experiments. Data from GF mice represent 1 independent experiment.

### Bleomycin model of pulmonary fibrosis, mouse tissue collection/processing, and hydroxyproline assay.

Pulmonary fibrosis was induced with oropharyngeal bleomycin as previously described in SPF and GF facilities (46). Control mice where applicable received identical volumes of saline instillation. Tissue collection and processing were performed as previously described (13). Collagen deposition was measured using a hydroxyproline assay as described previously (47, 48).

### DNA extraction and 16S rRNA gene sequencing.

DNA was isolated using QIAGEN DNeasy Kit and previously published protocol; the V4 region of the bacterial rRNA gene was amplified and sequenced using previously published protocols (13, 49). In brief, the V4 region of the 16S rRNA gene was amplified using published primers (50) and the dual indexing sequencing strategy developed by the laboratory of Patrick D. Schloss (51). Sequencing was performed using the Illumina MiSeq platform, using a MiSeq Reagent Kit V2 (500 cycles), according to the manufacturer’s instructions with modifications found in the Schloss standard operating procedure. Primary PCR cycling conditions were 95°C for 2 minutes, followed by 20 cycles of touchdown PCR (95°C 20 seconds, 60°C 20 seconds and decreasing by 0.3°C each cycle, 72°C 5 minutes), then 20 cycles of standard PCR (95°C for 20 seconds, 55°C for 15 seconds and 72°C for 5 minutes), and finished with 7°C for 10 minutes.

### Preparation of single-cell suspensions from lung tissue and flow cytometry.

Collagenase digestion of mouse lung tissue and flow cytometry were performed as previously described (25). See the Supplemental Methods for details.

### BAL and cytokine and IgM measurements.

BAL on experimental mice was performed as previously described (52). Pulmonary cytokine measurements were performed on murine lung homogenate using a Luminex platform (MilliporeSigma) as previously described (13). IgM was measured using Bethyl Laboratories catalog E99-101 mouse IgM ELISA kit per manufacturer’s instructions. BAL fluid was diluted to 1:1,000.

### FMT.

FMT was performed based on previously described methods (28). FMT solution was prepared fresh immediately before each FMT. A total of 8 fecal pellets from healthy age-matched C57BL/6J or C57BL/6NCrl mice were gently homogenized in 1 mL phosphate buffered saline (PBS). Homogenate was centrifuged at 3,000 rpm at room temperature for 30 seconds to remove debris. Supernatant was diluted in PBS to a volume of 2.2 mL, and the solution was given in 200 μL aliquots via oral gavage to recipient mice. SPF mice received an oral gavage once a week for 3 weeks. GF mice received 1 oral gavage.

### Gene expression by RNA-Seq.

RNA was extracted via RNeasy column kit (QIAGEN) following the manufacturer’s directions. Only samples with an RNA integrity number of 6 or higher as assessed by analysis on an Agilent Bioanalyzer were considered for RNA-Seq analysis. Sequencing was performed by GENEWIZ using a standard sequencing pipeline. In short, 30 million to 50 million read depth was achieved, and sequences were trimmed using Trimmomatic v.0.36. STAR aligner v.2.5.2b was used to map reads to the Ensembl mouse reference genome, after which unique gene hit counts of interexon regions were determined using FeatureCounts from the Subread package v.1.5.2. Differential expression analysis was conducted using DeSEQ2 in R v.3.6. A cutoff of ±1.5-fold change with an FDR ≤ 0.1 was used to identify differentially expressed genes. A full list of differentially expressed genes is available in the supplement (Supplemental Table 3). PCA and PERMANOVA were conducted in Python 3.7 using the Scitkitlearn learn package. GSEA was performed using the MSigDB immune signatures database (53).

### Community shotgun metagenomics.

Extracted DNA from 4 fecal samples, 1 sample per group, was sequenced with the metagenomic shotgun approach for a deeper resolution of the microbial community. Library preparation and sequencing were handled by the Advanced Genomics Core at the University of Michigan. Briefly, DNA was assessed using Qubit dsDNA High Sensitivity Assay (Thermo Fisher Scientific) and TapeStation genomic DNA reagents (Agilent). Libraries were prepared using NEBNext Ultra II FS DNA Library Prep Kit for Illumina (New England Biolabs) per manufacturer’s protocol with 400 ng DNA input. Libraries were checked for quantity by Qubit dsDNA High Sensitivity Assay (Thermo Fisher Scientific) and quality by LabChip (PerkinElmer) and run on a MiSeq to verify balance before being sequenced on the Illumina NovaSeq S4 paired-end 150 bp, according to manufacturer’s recommended protocols. Over 130 million paired-end 150 bp reads were subjected to quality control (QC) procedures. Based on an initial estimate of read qualities with FastQC (v0.11.9, https://www.bioinformatics.babraham.ac.uk/projects/fastqc/), reads were subjected to 1) adapter sequence detection and trimming, 2) low-quality (Phred ≤ 30) base trimming, and 3) short read pair (pairs where any read < 50 bp) filtering using TrimGalore (v0.6.7, https://www.bioinformatics.babraham.ac.uk/projects/trim_galore/) and Cutadapt (v4.0) (54). Reads containing tandem repeats and mouse host reads were removed from the sets using the KneadData pipeline (v0.10.0) (55). Reads were tested for any remaining sequence overrepresentation with SNIKT (v0.4.2) (56) and were resolved with bulk trimming by 10 bp from 5′ and 2 bp from 3′ ends using SNIKT. QC reports were generated with FastQC and compiled with MultiQC (v1.12) (56). GNU Parallel (v20220222) (57) was used throughout the informatic analysis for parallelization of procedures over samples. Microbial community estimation was performed using quantification of clade-specific marker gene fragments in the microbial reads. MetaPhlAn (v3.0.14) (58) was run with the v30 ChocoPhlAn marker gene database to profile the microbial community. Following the observation that over 87% of the community remained uncharacterized, MetaPhlAn was repeated in local mode, improving the estimate of uncharacterized fraction to over 63%. The abundance profiles were visualized with Pavian (v1.0) (59). Individual abundance profiles were merged at genus and species levels and visualized as heatmaps with utilities in MetaPhlAn.

### Statistics.

16S sequence data were processed and analyzed using mothur version 1.42.3 according to the standard operating procedure for MiSeq sequence data using a minimum sequence length of 250 bp (51). For each experiment and sequencing run, a shared community file and a phylotyped (genus level grouping) file were generated using OTUs binned at 97% identity generated using the dist.seqs, cluster, make.shared, and classify.otu commands in mothur. OTU numbers were arbitrarily assigned in the binning process and are referred to in association with their most specified level of taxonomy. Classification of OTUs was carried out using the mothur implementation of the Ribosomal Database Project (RDP) Classifier and the RDP taxonomy training set 14 (Trainset14_032015.rdp), available on the mothur website. The *vegan* and *mvabund* package and a customized machine learning random forest ensemble tool were used to further analyze community sequencing data. GLMs were used for multivariate analysis (*mvabund*) with *P* statistics generated for each response variable after accounting for multiple comparisons with a step-down resampling procedure. For relative abundance and ordination analysis, samples were normalized to the percentage of total reads, and we restricted analysis to OTUs that were present at greater than 1% of the sample population. All OTUs were included in diversity analysis. Direct community similarity comparisons were performed using the Bray-Curtis similarity index. We performed ordinations using PCA on Hellinger-transformed OTUs, with family or phylum tables generated using Euclidean distances. We determined significance of differences in community composition using PERMANOVA (adonis) with 10,000 permutations using Euclidean distances. We compared means via 2-tailed *t* test, 1-way ANOVA, paired 2-tailed *t* test or Mann-Whitney test, or Wilcoxon’s rank-sum tests where appropriate. Data are presented as mean ± SEM. All analysis was carried out in R version 4.0.0.

### Study approval.

All animal experiments reported in this manuscript were approved by the Institutional Animal Care & Use Committee at the University of Michigan. All laboratory animal care policies followed the Public Health Service Policy on Humane Care and Use of Laboratory Animals at the University of Michigan.

### Data availability.

16S and metagenome sequencing data are available via the NCBI Sequence Read Archive BioProject ID PRJNA859868. RNA-Seq data are available from NCBI GEO number GSE209784. Data are available in the Supporting Data Values file. Applicable code is available upon request to the corresponding author.

## Author contributions

DNO, SJG, and PR conceived the studies; carried out experiments, analysis, and interpretation of data; and wrote the manuscript. JHL carried out experiments and analyzed and interpreted data. JS and SA carried out experiments. LHK and BBM collected data and contributed to manuscript drafting. GBH contributed to manuscript drafting. All authors approved of the final draft of the manuscript.

## Supplementary Material

Supplemental data

Supporting data values

## Version 1 11/28/2023

In-Press Preview

## Version 2 01/09/2024

Electronic publication
