# Supplementary material for: Horizontal transmission of gut microbiota attenuates mortality in lung fibrosis
Source: JCI Insight. 2023 Nov 28;9(1):e164572. doi: 10.1172/jci.insight.164572 (PMC10911107; doi:10.1172/jci.insight.164572)
Supplement: Supplemental data [file jciinsight-9-164572-s008.pdf]

Supplement Title Page:

"Horizontal transmission of gut microbiota attenuates mortality in lung fibrosis"

Stephen J Gurzynski[1], Jay H Lipinski[2], Joshua Strauss[2], Shafiul Alam[2], G Huffnagle, Piyush Ranjan[2], Lucy H Kennedy[3], Bethany B Moore[1,2], and David N O'Dwyer[2]

1. Dept of Microbiology and Immunology, University of Michigan, Ann Arbor, MI
2. Division of Pulmonary and Critical Care Medicine, University of Michigan Medical School, Ann Arbor, MI
3. Unit for Laboratory and Animal Medicine, University of Michigan, Ann Arbor, MI

Corresponding Author:

David N O'Dwyer
4049 BSRB
Zina Pitcher Pl
Ann Arbor, 48109
MI, USA

Supplement Fig.S1

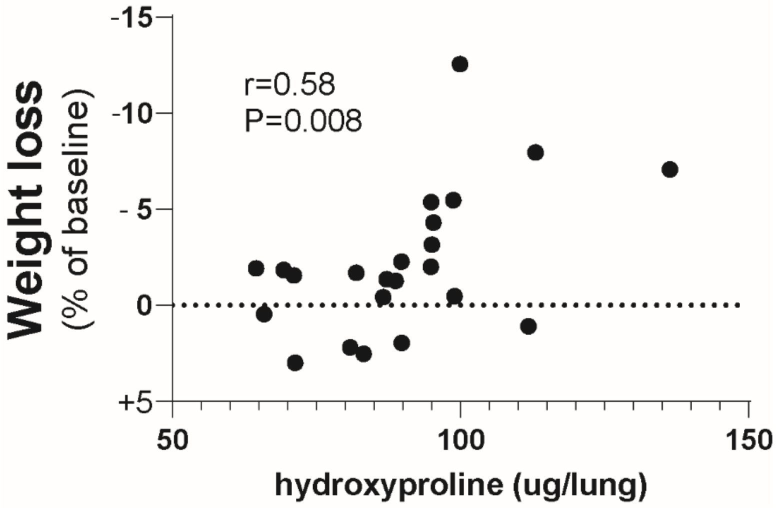

Percent weight loss day 4 post bleomycin in surviving mice correlates with lung collagen content day 21 post bleomycin (Spearman correlation)

Supplement Fig.S2

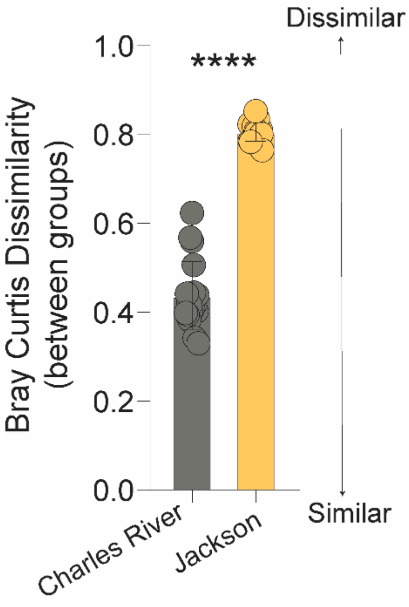

Bray Curtis dissimilarity scores between BL/Cr and BL/6J mice on arrival from barrier facility. Calculated at the operational taxonomic unit (OTU) level of classification. (n=20,****P<0.0001)

Supplemental Table S1

NB-GLM result table of analysis of taxa (family) differentially abundant between CR and JAX cohorts on arrival from barrier facilities.

Family	P value
Clostridiales_unclassified	0.001
Erysipelotrichaceae	0.001
Lactobacillaceae	0.001
Verrucomicrobiaceae	0.001
Bifidobacteriaceae	0.002
Anaeroplasmataceae	0.003
Deferribacteraceae	0.004
Ruminococcaceae	0.02

Supplemental Fig.S3

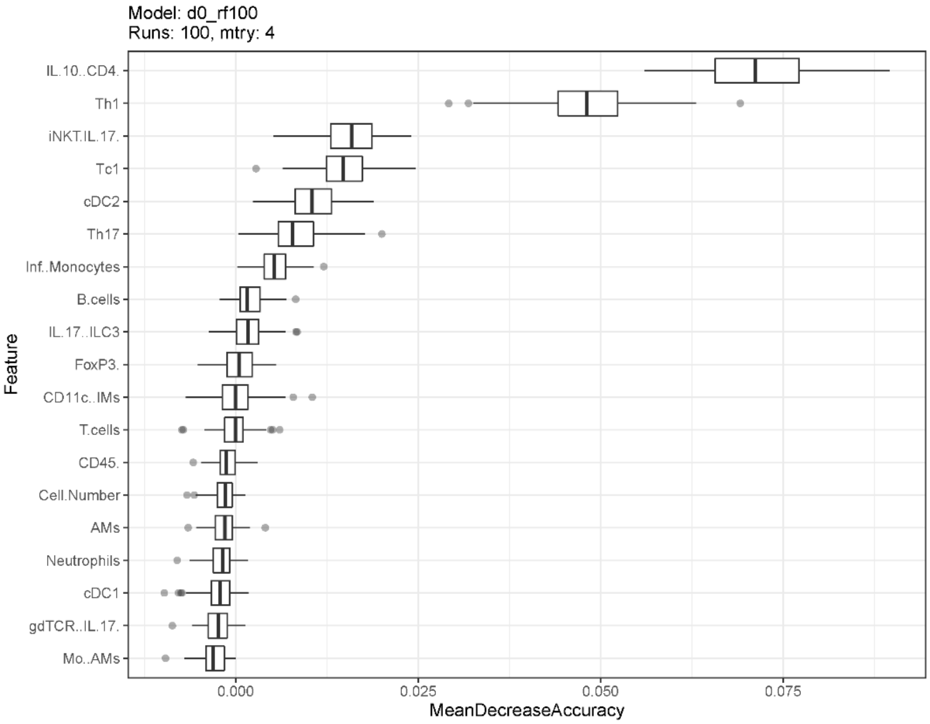

Random Forest machine learning ensemble technique to identify significantly increased leukocyte cell type in the lungs of C57BL/6Cr and BL/6J mice at baseline and untreated. CD4+ IL-10+ and Th1 lymphocytes are found with increased frequency in BL/6Cr mice.

Supplemental Fig.S4

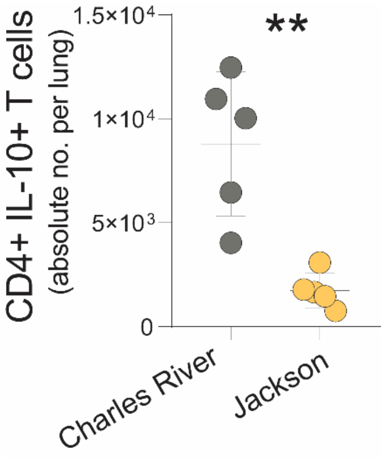

Supplemental Fig.S5

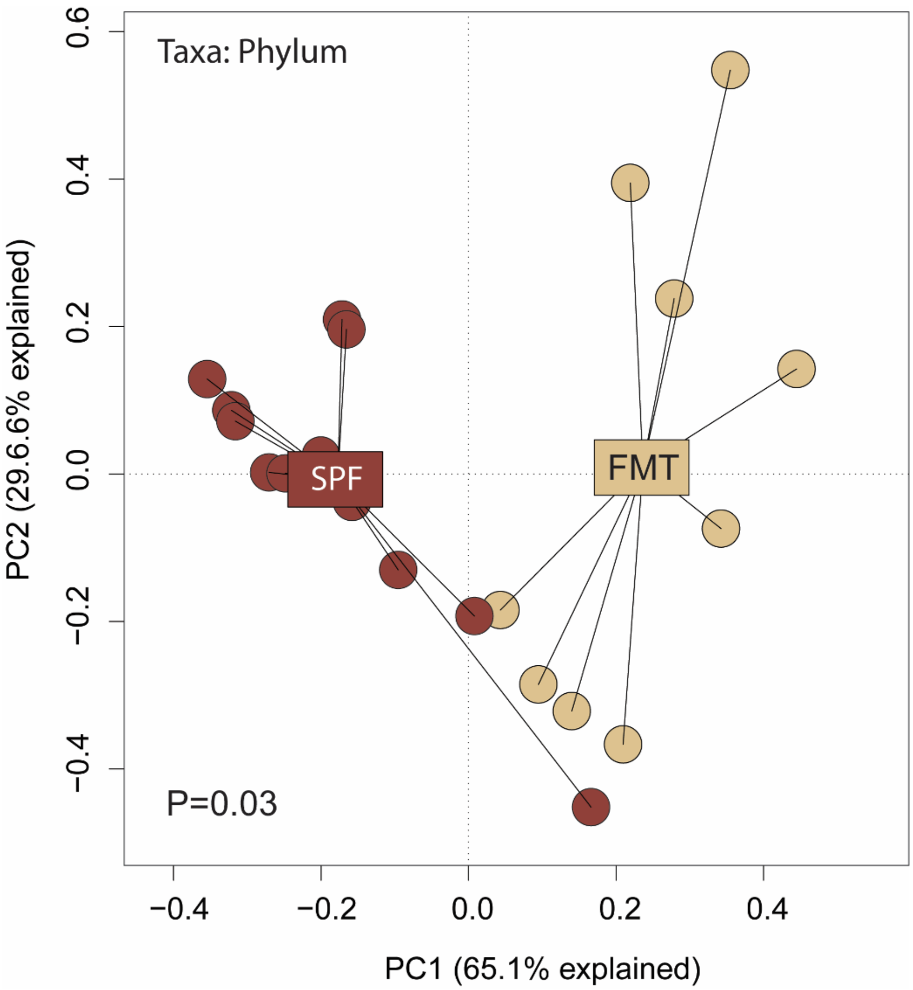

Bray Curtis dissimilarity scores form 16S gut data on day 0 of cohousing. ****P<0.0001.

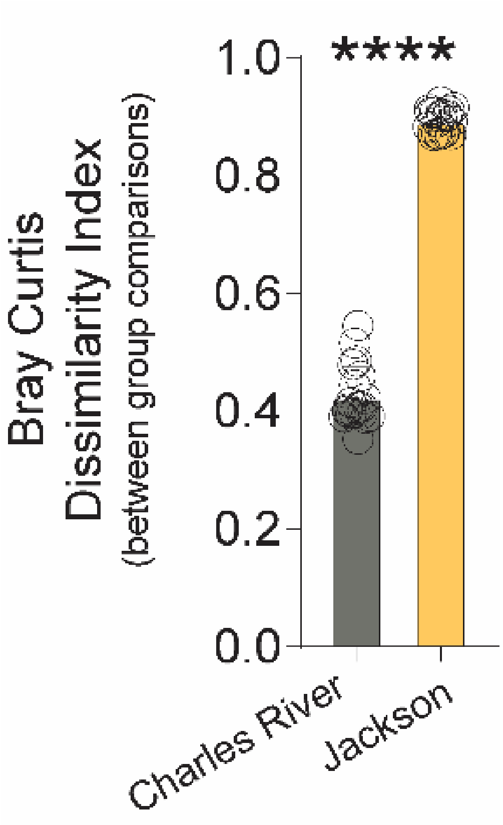

Supplemental Fig.S6

Supplement Table S2.

NB-GLM analysis of JAX and CR differentially abundant taxa (family) day 0 before cohousing

Family	P value
Bacteroidaceae	0.001
Bacteroidales_unclassified	0.001
Bacteroidetes_unclassified	0.001
Bifidobacteriaceae	0.001
Deferribacteraceae	0.001
Desulfovibrionales_unclassified	0.001
Erysipelotrichaceae	0.001
Lactobacillaceae	0.001
Prevotellaceae	0.001
Rikenellaceae	0.001
Sutterellaceae	0.001
Verrucomicrobiaceae	0.001
Peptostreptococcaceae	0.002
Anaeroplasmataceae	0.003
Enterobacteriaceae	0.003
Clostridiales_unclassified	0.004
Clostridiaceae_1	0.018
Eubacteriaceae	0.028

Overlapping taxa highlighted

Table S3. Differentially expressed genes in comparative assessment between Jackson and Charles River original groups.

**symbol**	**baseMean**	**log2FoldChange**	**lfcSE**	**stat**	**pvalue**	**padj**
Gm16867	17.153	6.963	2.019	3.450	0.001	0.077
ND3	98.957	5.521	1.064	5.190	0.000	0.004
Sycp1	11.994	4.874	1.140	4.277	0.000	0.028
Afm	8.859	4.755	1.329	3.578	0.000	0.063
Myh2	7.631	3.843	1.030	3.733	0.000	0.048
Gm21451	15.055	3.564	0.940	3.794	0.000	0.041
Saa3	1603.139	3.366	0.812	4.148	0.000	0.028
Tnfsf18	13.311	3.056	0.744	4.109	0.000	0.028
Gm15446	77.565	2.860	0.831	3.441	0.001	0.079
Ttn	133.644	2.811	0.790	3.556	0.000	0.066
Gm43059	16.391	2.703	0.723	3.737	0.000	0.048
Nr6a1os	14.687	2.483	0.729	3.409	0.001	0.084

Gm43111	11.240	2.388	0.616	3.877	0.000	0.037
Fga	9.814	2.329	0.696	3.347	0.001	0.092
Lrmda	136.740	2.227	0.444	5.017	0.000	0.006
Crabp2	9.439	2.207	0.636	3.469	0.001	0.077
Gm43775	31.545	2.189	0.557	3.931	0.000	0.034
Dcstamp	54.057	2.063	0.532	3.876	0.000	0.037
Neb	53.940	2.001	0.555	3.608	0.000	0.061
Cd5l	99.514	1.966	0.517	3.803	0.000	0.041
Gm37788	18.116	1.896	0.550	3.445	0.001	0.078
Marco	531.108	1.874	0.559	3.350	0.001	0.091
Inhba	114.533	1.676	0.439	3.814	0.000	0.041
Zfp811	20.155	1.470	0.430	3.416	0.001	0.083
Crybb3	40.934	1.468	0.425	3.458	0.001	0.077
Gm21972	40.426	1.463	0.424	3.447	0.001	0.078
Hal	78.321	1.402	0.337	4.160	0.000	0.028
Prg4	284.388	1.394	0.353	3.949	0.000	0.033
Gm44763	13.454	1.377	0.389	3.541	0.000	0.068
Zfp993	28.486	1.346	0.389	3.456	0.001	0.077
Ffar2	136.743	1.330	0.313	4.245	0.000	0.028
Vwc2	61.445	1.294	0.372	3.480	0.001	0.076
Tmem213	55.597	1.217	0.363	3.356	0.001	0.091
Shisa8	46.824	1.203	0.336	3.582	0.000	0.063
Fabp4	702.041	1.157	0.316	3.661	0.000	0.054
Dio2	30.249	1.130	0.340	3.324	0.001	0.097
Smim36	135.637	1.046	0.265	3.944	0.000	0.033
Nfe2	131.265	1.016	0.241	4.217	0.000	0.028
Dok2	550.372	0.979	0.252	3.887	0.000	0.037
Cfp	1042.025	0.951	0.252	3.781	0.000	0.043
Treml2	88.362	0.947	0.258	3.672	0.000	0.053
Prkcb	432.105	0.923	0.257	3.591	0.000	0.063
Itgam	531.036	0.904	0.257	3.515	0.000	0.070
Batf3	144.065	0.875	0.238	3.676	0.000	0.053
Slc7a11	197.461	0.873	0.245	3.567	0.000	0.065
Nnmt	530.986	0.861	0.220	3.912	0.000	0.036
Ccl17	194.488	0.838	0.210	3.984	0.000	0.032
Zfp268	75.898	0.836	0.202	4.136	0.000	0.028
S100a4	788.510	0.819	0.227	3.600	0.000	0.062
Pdlim4	367.526	0.815	0.208	3.918	0.000	0.035
Relt	232.782	0.798	0.196	4.069	0.000	0.030
Cyp4f18	413.294	0.770	0.159	4.856	0.000	0.006
Cd52	1059.402	0.759	0.214	3.550	0.000	0.066
Fut7	63.387	0.750	0.208	3.607	0.000	0.061
Psme2b	530.875	0.738	0.208	3.551	0.000	0.066

Ubash3b	707.187	0.734	0.174	4.218	0.000	0.028
Slc43a1	136.005	0.734	0.212	3.457	0.001	0.077
Mcemp1	623.786	0.716	0.201	3.561	0.000	0.065
Ltc4s	512.546	0.700	0.159	4.414	0.000	0.020
Adgrd1	1170.694	0.699	0.187	3.739	0.000	0.048
Fam133b	1146.779	0.697	0.211	3.309	0.001	0.099
Cmah	974.519	0.689	0.161	4.271	0.000	0.028
Trim29	186.824	0.687	0.203	3.384	0.001	0.087
AI662270	426.670	0.680	0.182	3.729	0.000	0.048
Emp3	1547.085	0.678	0.202	3.358	0.001	0.091
Fcer1g	1750.312	0.672	0.168	3.994	0.000	0.031
Baz1a	1295.960	0.658	0.192	3.435	0.001	0.079
9230114K14Rik	103.528	0.655	0.194	3.369	0.001	0.090
Spi1	1815.830	0.649	0.178	3.639	0.000	0.058
Mgll	4297.842	0.640	0.189	3.393	0.001	0.086
Hvcn1	1136.039	0.639	0.183	3.487	0.000	0.075
Ccdc88a	927.911	0.639	0.161	3.969	0.000	0.033
Tspan32	284.364	0.634	0.155	4.086	0.000	0.028
Socs3	2160.686	0.607	0.183	3.313	0.001	0.098
Arhgdib	2825.924	0.606	0.174	3.488	0.000	0.075
Myo15b	639.682	-0.631	0.153	-4.121	0.000	0.028
2610528A11Rik	173.835	-0.636	0.158	-4.022	0.000	0.031
Pla2g4f	271.797	-0.637	0.173	-3.678	0.000	0.053
Chdh	119.000	-0.642	0.166	-3.858	0.000	0.039
Thbs3	4708.253	-0.653	0.172	-3.802	0.000	0.041
Col13a1	988.959	-0.654	0.193	-3.381	0.001	0.088
Gpa33	460.513	-0.658	0.196	-3.358	0.001	0.091
Myo1a	589.559	-0.660	0.187	-3.535	0.000	0.068
Ctnnd2	314.346	-0.666	0.179	-3.723	0.000	0.048
Btnl6	202.216	-0.667	0.196	-3.400	0.001	0.084
Cdh17	675.785	-0.668	0.165	-4.047	0.000	0.031
Edn3	602.256	-0.669	0.178	-3.753	0.000	0.046
Apol10a	550.354	-0.673	0.169	-3.992	0.000	0.031
Fat2	191.416	-0.675	0.203	-3.320	0.001	0.097
Tcf21	1315.916	-0.676	0.179	-3.780	0.000	0.043
Tff3	313.027	-0.684	0.204	-3.358	0.001	0.091
B4galnt2	256.642	-0.684	0.188	-3.634	0.000	0.058
Zg16	1056.719	-0.707	0.203	-3.488	0.000	0.075
Bmp2	192.820	-0.713	0.185	-3.854	0.000	0.039
Mgam	252.956	-0.717	0.174	-4.127	0.000	0.028
Hhip	586.660	-0.717	0.203	-3.533	0.000	0.068
Duox2	304.729	-0.718	0.185	-3.885	0.000	0.037
Itgae	230.577	-0.719	0.187	-3.845	0.000	0.039

Eps8l3	107.070	-0.721	0.208	-3.470	0.001	0.077
Baiap3	151.378	-0.724	0.202	-3.578	0.000	0.063
B3galt5	413.898	-0.735	0.165	-4.455	0.000	0.020
Hnf4a	502.241	-0.736	0.194	-3.803	0.000	0.041
Adgrg7	205.648	-0.738	0.196	-3.767	0.000	0.044
Myh7	824.825	-0.771	0.192	-4.017	0.000	0.031
Saa1	563.730	-0.786	0.216	-3.633	0.000	0.058
Muc2	2138.469	-0.790	0.197	-4.011	0.000	0.031
Mep1a	123.566	-0.790	0.198	-3.991	0.000	0.031
Dgkq	207.978	-0.795	0.208	-3.828	0.000	0.040
Mgam2-ps	90.024	-0.802	0.236	-3.399	0.001	0.084
Dmbt1	4492.756	-0.804	0.164	-4.910	0.000	0.006
Retnlb	409.889	-0.839	0.218	-3.842	0.000	0.039
Ceacam20	101.182	-0.852	0.222	-3.839	0.000	0.039
Slc51b	92.175	-0.872	0.235	-3.718	0.000	0.049
Cap2	257.113	-0.877	0.191	-4.589	0.000	0.019
Tm4sf20	140.697	-0.883	0.216	-4.087	0.000	0.028
Adcy8	223.397	-0.916	0.239	-3.837	0.000	0.039
Ugt2b34	185.684	-0.925	0.207	-4.462	0.000	0.020
Nnt	1063.507	-0.981	0.237	-4.137	0.000	0.028
Dynlt1b	725.484	-0.994	0.294	-3.375	0.001	0.089
1810049J17Rik	110.969	-1.010	0.304	-3.319	0.001	0.097
Gm19461	65.113	-1.072	0.304	-3.527	0.000	0.069
Cnr1	55.486	-1.093	0.284	-3.848	0.000	0.039
Gzmb	49.465	-1.140	0.318	-3.586	0.000	0.063
Slc9a3	80.160	-1.209	0.324	-3.726	0.000	0.048
Efcab7	145.000	-1.224	0.304	-4.024	0.000	0.031
Camk2b	307.875	-1.269	0.320	-3.966	0.000	0.033
Kcnf1	146.121	-1.343	0.339	-3.961	0.000	0.033
Lyz1	17879.359	-1.451	0.430	-3.373	0.001	0.089
Slc47a1	22.399	-1.561	0.457	-3.418	0.001	0.083
Cyp1a1	92.423	-1.914	0.519	-3.684	0.000	0.053
Nell1	18.496	-2.382	0.673	-3.541	0.000	0.068
Gm38293	7.253	-3.288	0.952	-3.453	0.001	0.077
1700021J08Rik	8.020	-3.340	0.922	-3.623	0.000	0.059
Mid1-ps1	10.941	-3.505	0.865	-4.054	0.000	0.031
1700007J10Rik	5.607	-4.212	1.078	-3.906	0.000	0.036
Gm43403	3.692	-4.379	1.242	-3.527	0.000	0.069
Try5	14.092	-18.613	4.473	-4.161	0.000	0.028
Try4	14.462	-18.912	4.458	-4.242	0.000	0.028

Supplemental Fig.S7

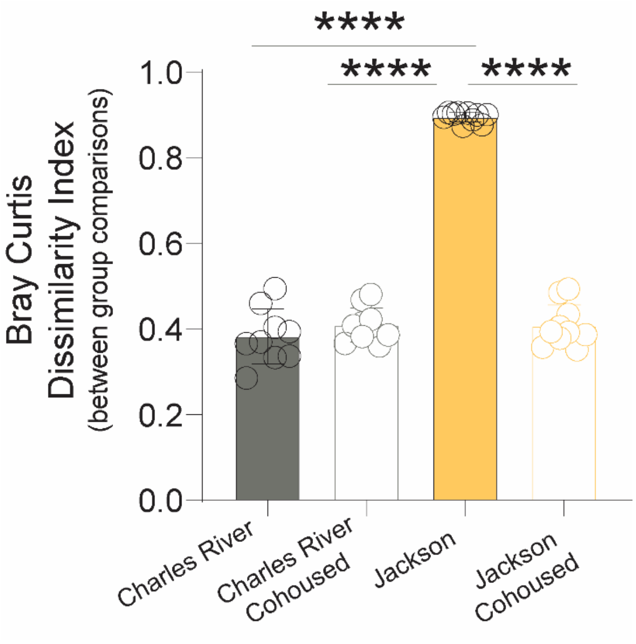

Bray Curtis dissimilarity scores form 16S gut data on day 21 of cohousing. ****P<0.0001.

Supplemental Fig.S8

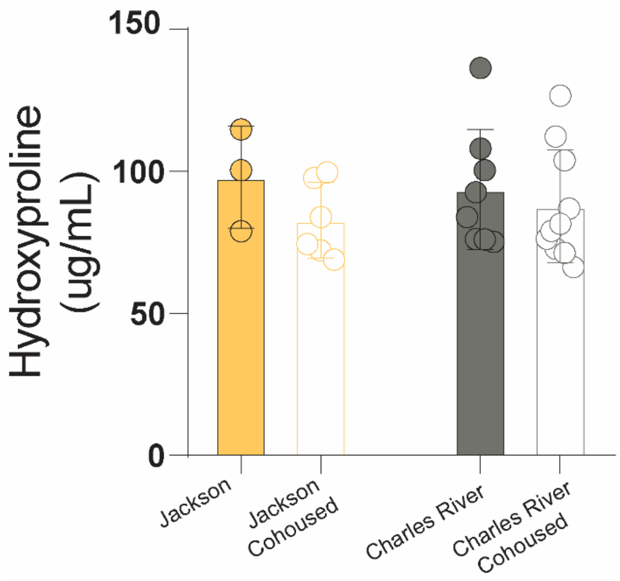

Lung collagen content in original BL/6CR and BL/6J and mixed BL/6Cr and BL/6J experimental mice

Supplement Table S4.

NB-GLM model results. Changes in taxa after 21 Days of cohousing across all 4 groups

Family	P value
Bacteroidaceae	0.001
Bacteroidales_unclassified	0.001
Bacteroidetes_unclassified	0.001
Desulfovibrionales_unclassified	0.001
Erysipelotrichaceae	0.001
Prevotellaceae	0.001
Rikenellaceae	0.001
Clostridiaceae_1	0.003
Anaeroplasmataceae	0.011
Deferribacteraceae	0.048

Supplemental Fig.S9

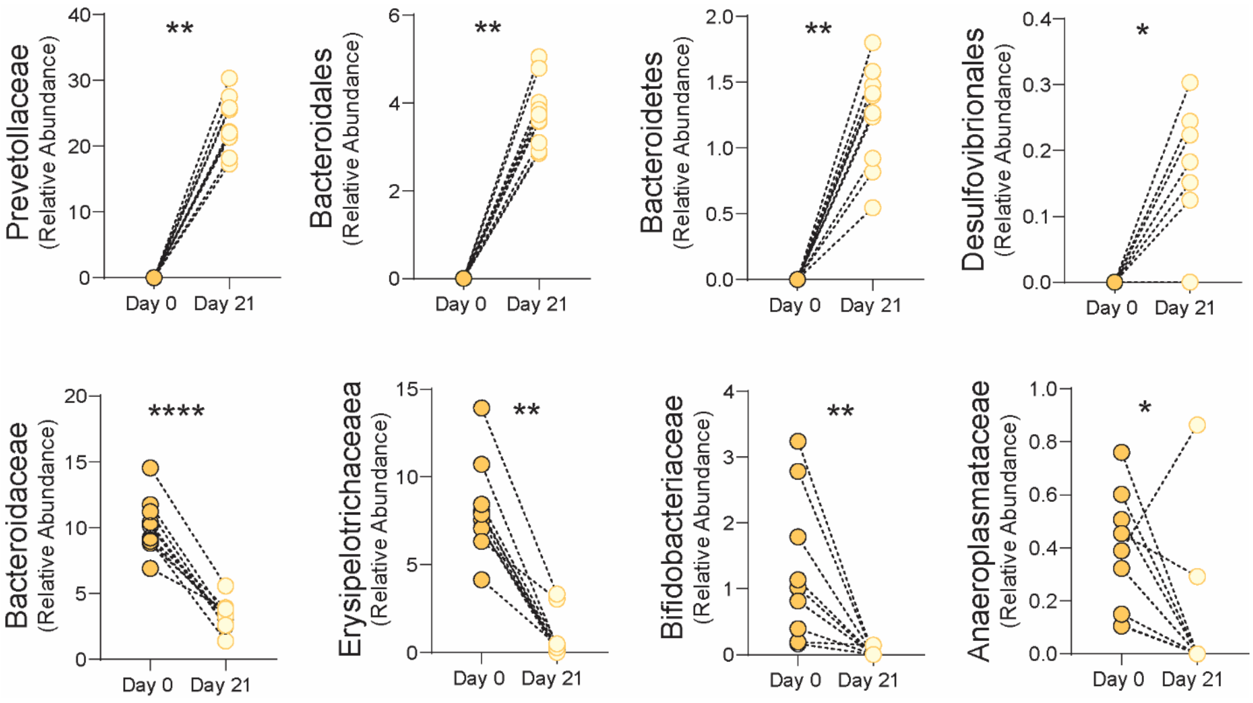

Relative abundance of taxa that significantly change over time form day 0 to day 21 in C57BL/6J mice cohoused with CR mice. Paired T test or Wilcoxon matched rank test. ****P<0.0001, **P<0.01, *P<0.01

Supplemental Fig.S10

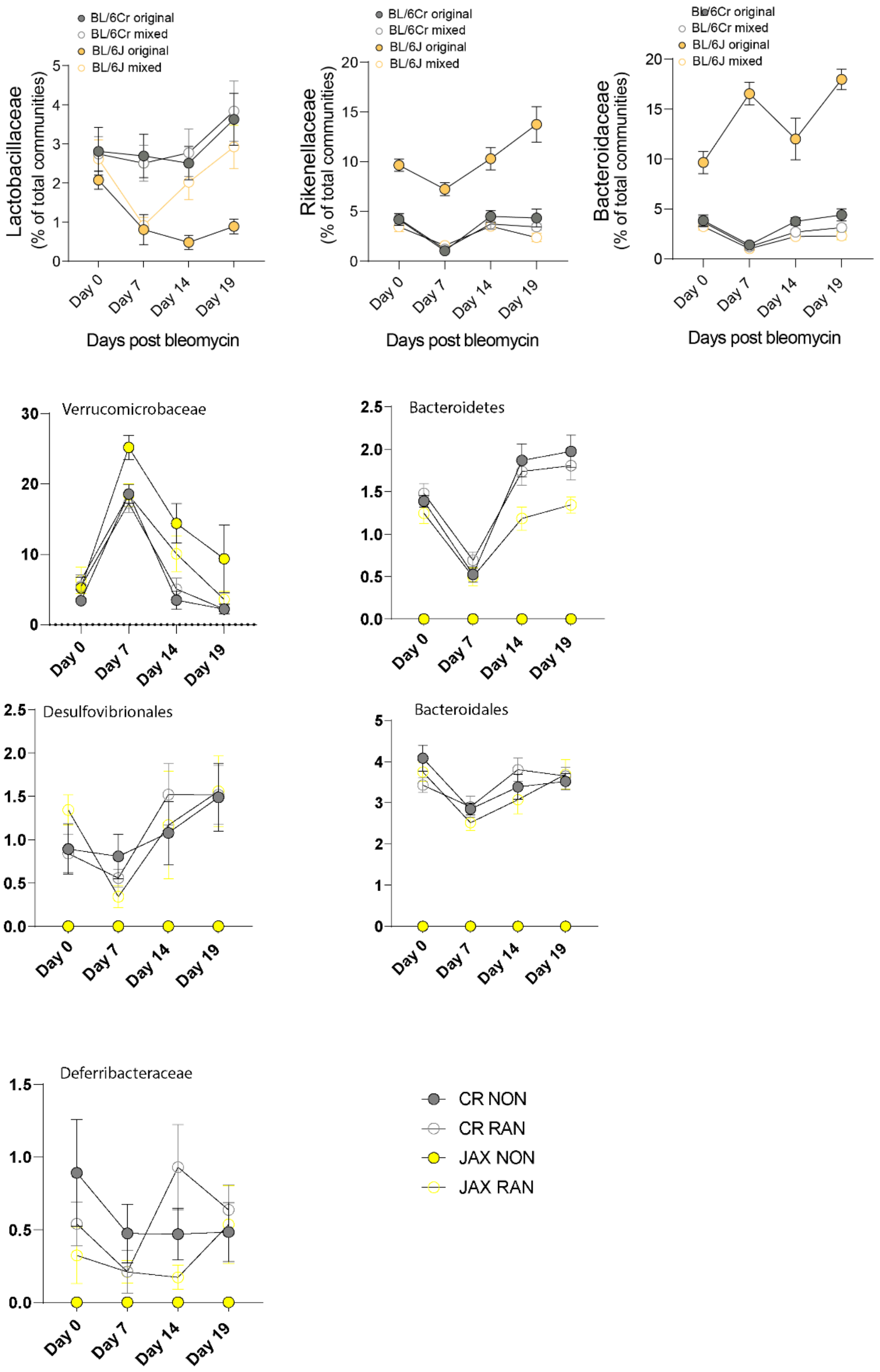

Changes in taxa over time post bleomycin in C7BL/6J and C57BL/6Cr mice.

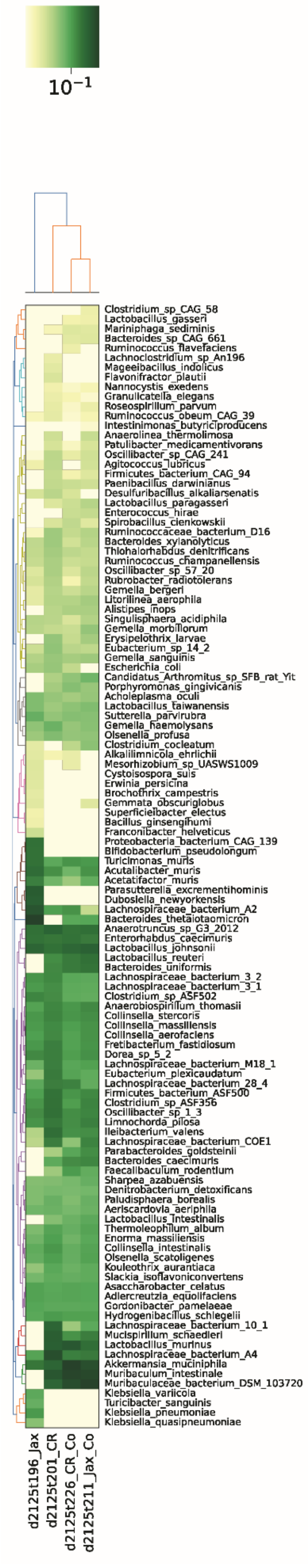

Supplemental Fig.S11

Heatmap of data generated from shotgun sequencing of fecal DNA from C57BL/6 sub strain mice and catalogued using genetic makers by MetaPhlAn

Supplemental Methods

Preparation of single cell suspensions and flow cytometry of leukocyte populations. Groups of mice were harvested via CO_2 asphyxiation at the indicated time points and perfused through the left ventricle of the heart with 3-5 mL of PBS. Lungs were resected and minced with scissors before digestion in complete DMEM media containing 10% FBS, 1 mg / mL Collagenase A (Roche), and 2000 u of DNAse I (Sigma) at 37°C for 45 minutes in a shaking incubator. Lung minces were further disrupted through a 10 mL syringe and leukocytes were isolated by centrifugation of the disrupted cell pellets through a 20% Percol solution before a final filtering through a 10-micron nytex filter. After isolation, cells were enumerated, and viability was assessed at > 80% by trypan blue staining.

To perform intracellular cytokine staining**,** equal numbers of cells from each animal ($5x10^6$ cells per sample) were incubated for 4 h in complete DMEM media containing 10% FBS, PMA (10 ng/mL), ionomycin (10μM), and GolgiStop reagent (BD Biosciences). Following stimulation $1x10^6$ cells were stained for flow cytometry with the following antibodies. PE-Cy7–IFN-γ. (clone XMG1.2), FITC-CD3 (clone 17A2), BV510-CD45 (clone 30-F11) purchased from BD Biosciences. APC-Cy7-IL17A (clone TC11-18H10.1), BV570-CD8a (clone 53-6.7), AF700-CD90.2 (Thy1.2 clone 30-H12), BV421-CD4 (clone GK1.5), PE-IL-4 (clone 11B11) purchased from BioLegend. PE-eFluor 610-FoxP3 (clone FJK-16s purchased from eBioscience, Thermo Fisher Scientific. APC-IL-10 (clone JES5-16E3) purchased from Invitrogen.

Flow cytometry data was collected on a BD Fortessa cytometer and analyzed in FlowJo v. 10.5. Dimensional reduction of flow cytometry data was performed in Flow Jo using the uMAP plugin v3.1
